# Regulatory mutation accumulation drives multi-trait phenotypic expansion in silver-adapted *Escherichia coli*

**DOI:** 10.1128/spectrum.00041-26

**Published:** 2026-10-12

**Authors:** Theresa D. Jones, Anuoluwapo O. Anele, Ishan J. Patel, Bryce J. Middleton, Leah M. Worrill, Jacquelle A. R. Joseph-Lainez, Brittany R. Sanders, Joseph L. Graves, Misty D. Thomas

**Affiliations:** 1 Department of Biology, North Carolina Agricultural and Technical State University 3616, Greensboro, North Carolina, USA; Illinois State University, Normal, Illinois, USA

**Keywords:** pleiotropy, transcriptional rewiring, regulatory mutations, gene expression profiling, two-component response systems, adaptation, experimental evolution

## Abstract

Predicting when adaptive mutations produce narrow, condition-specific effects versus coordinated multi-trait phenotypic expansion remains a central challenge in evolutionary biology. Two-component response systems (TCRSs) play a key role in bacterial environmental sensing and transcriptional regulation, yet how regulatory variation shapes broader adaptive outcomes remains poorly understood. Here, we reanalyze and integrate our previously published experimental evolution, whole-genome sequencing, and transcriptomic data with new high-throughput phenotypic profiling, and competitive fitness assays to examine how regulatory context influences multi-trait responses during bacterial adaptation. Using silver-adapted *Escherichia coli* populations, we show that independently evolved lineages carrying *cusS* (a TCRS with CusR that regulates copper/silver efflux) mutations, along with additional regulatory mutations, exhibit broader phenotypic expansion across metabolic and stress-response conditions, whereas engineered single *cusS* mutations produce constrained phenotypic responses. Comparative transcriptomic analyses reveal that evolved populations display expanded engagement of functional pathways relative to engineered mutants, consistent with increased regulatory diversification. However, competitive fitness assays against an engineered single mutant revealed that this phenotypic expansion was not uniformly adaptive: four of six evolved lineages fell below parity against the engineered mutant, while two lineages, despite comparable phenotypic breadth, did not. Together, these results indicate that multi-trait phenotypic expansion during adaptation is associated with regulatory mutation accumulation and transcriptional remodeling rather than with isolated adaptive mutations, but that this expansion does not reliably predict competitive fitness outcome. More broadly, our findings highlight the importance of regulatory architecture in shaping evolutionary trajectories and underscore the need for direct fitness measurements when interpreting coordinated phenotypic responses in microbial evolution.

**IMPORTANCE:**

Bacteria constantly encounter stresses, such as antibiotics and toxic metals, and must adapt to survive. While individual genetic changes can help bacteria tolerate stress, it remains unclear why some adaptations affect only a single trait while others reshape many aspects of physiology, and whether these broader changes make bacteria more competitive or simply reflect disorganized regulation. Here, we combined evolutionary experiments, genomic and transcriptomic data, phenotypic profiling, and head-to-head competition assays to show that broad changes in bacterial behavior are associated with accumulation of regulatory mutations rather than single adaptive changes, but do not always confer a real competitive advantage. Our results show that regulatory network structure shapes how widely adaptation impacts metabolism, stress tolerance, and cellular function, while also showing that phenotypic breadth alone does not reliably predict fitness. These findings advance our understanding of bacterial evolution and may inform efforts to manage microbial responses to clinical stressors.

## INTRODUCTION

Understanding how genetic variants impact organismal fitness remains a cornerstone of evolutionary biology (1). Microorganisms, in particular, display extraordinary adaptability to fluctuating environments, yet the mechanisms by which genetic variation reorganizes cellular function to support complex adaptive outcomes are still poorly understood (2–5). A major component of this regulatory flexibility is mediated through two-component response systems (TCRSs), which couple environmental sensing to transcriptional reprogramming (6–9). These systems, composed of a membrane-bound histidine kinase (HK) and cognate response regulator (RR), act as primary signal-processing modules that rapidly reshape gene expression in response to external cues. Through this architecture, TCRSs influence diverse physiological processes, including metabolism, stress tolerance, virulence, and membrane remodeling. However, while they play a central role in environmental sensing, it remains unclear how broad, coordinated multi-trait adaptive responses are associated with regulatory context and mutation accumulation across evolutionary timescales (6, 10, 11).

Although TCRSs have been extensively studied in the context of short-term acclimation and stimulus-response signaling, their role in longer-term evolutionary change remains unresolved (10, 12). In particular, it is unclear when regulatory mutations in these systems are sufficient to alter broader cellular programs versus when they produce narrow or condition-specific effects. This uncertainty limits our ability to predict how bacteria will respond to selection pressures such as antimicrobials or nutrient limitation. Emerging evidence suggests that TCRS mutations are associated with broader transcriptional and physiological changes and, under some conditions, enable multi-level shifts in cellular performance (13, 14). However, the conditions under which such regulatory changes are associated with broad versus restricted adaptive outcomes remain largely unknown, representing a critical barrier to understanding how signaling variation is translated into evolutionary potential (15).

Our prior work on silver-adapted populations carrying mutations in the histidine kinase *cusS* established a model for examining how regulatory mutations are associated with downstream physiological variation (7, 16). In those studies, we recombinantly engineered four naturally occurring chromosomal point mutations (*cusS*: R15L, T14P, T17P, and N279H [17]) that arose during experimental evolution for adaptation to silver nitrate into the ancestral background (18, 19). We showed that each variant constitutively expressed the *cusCFBA* copper/silver efflux pump while also producing a distinct gene expression and fitness profiles across environments, consistent with strong genotype-by-environment (G × E) interactions (7, 16). RNA-seq revealed that while all mutants upregulated *cusS/cusR* and the downstream *cusCFBA* efflux operon, they diverged substantially in secondary transcriptional responses, indicating that the phenotypic consequences of *cusS* variation may extend beyond metal efflux alone.

In the subsequent study, we re-examined the silver-adapted mutant (SAM) populations that emerged from the same evolutionary experiment (19). Whole-genome sequencing revealed that three of the six SAM lineages that could be successfully revived from frozen stocks had accumulated additional regulatory mutations (*ompR*, *rho*, *fur*) alongside *cusS* (R15L), whereas the remaining three were silver resistant but lacked any *cusS* mutations (16). These findings indicated that changes in *cusS* alone are insufficient to explain the full evolutionary response and that additional regulatory interactions shape how adaptation manifests across environments. However, while these studies implicated regulatory epistasis in expanding adaptive potential, the extent to which such combinations produce broader multi-trait phenotypes (i.e., pleiotropic enhancement) remained unresolved (16). This motivated a direct comparison between engineered single-mutant backgrounds and independently evolved silver-adapted populations to determine how regulatory context influences multi-trait phenotypic outcomes.

In this study, we distinguish classical genetic pleiotropy, defined as a single locus influencing multiple traits (20–22), from systems-level pleiotropic expansion, which we define here, for the purposes of this study, as the coordinated emergence of multi-trait phenotypic divergence associated with regulatory mutation combinations and transcriptional context (23, 24). While individual mutations can contribute to such outcomes, our focus is on how combinations of regulatory perturbations reshape accessible phenotype space across metabolic and stress-response axes. In microbial systems, this means that a mutation initially selected for stress resistance may also reshape metabolic capacity or environmental tolerance more broadly. To determine whether regulatory changes in *cusS* alone are associated with coordinated multi-trait phenotypic expansion or whether broader regulatory background is required, we compared engineered single mutants to naturally evolved SAM populations across a diverse panel of metabolic and stress conditions, and further tested whether this phenotypic expansion corresponds to a competitive fitness advantage through direct competition assays. Using Biolog Gen III functional assays, we assessed functional utilization of 94 substrates and inhibitory conditions spanning carbon and nitrogen utilization, osmotic and pH stress, and antimicrobial compounds (25). These phenotypic data were then integrated with mutational and transcriptomic profiles from our prior studies (7, 16) to determine the breadth of multi-axis phenotypic responses beyond the original selection environment.

This integrated approach provides a framework for characterizing how regulatory mutation accumulation is associated with altered cellular performance across multiple physiological axes. By integrating genome-wide regulatory profiling, functional phenotyping, and direct competitive fitness assays, we examine patterns of coordinated regulatory changes and their functional consequences, rather than focusing on individual mutational events in isolation. In doing so, this work advances a system-level framework linking regulatory architecture to adaptive phenotype breadth, while also demonstrating that phenotypic breadth alone is not a reliable predictor of competitive fitness outcome.

## MATERIALS AND METHODS

### Bacterial strains and general culturing conditions

All strains used in this study were derived from *Escherichia coli* K-12 MG1655 (ATCC 10798), the ancestral background from prior experimental evolution with silver nitrate (19). The strain panel included the wild-type (WT), four engineered *cusS* single-point mutants (R15L, T14P, T17P, N279H) (17), and six silver-adapted mutant populations (SAM1–SAM6) isolated from evolved lineages (19). Glycerol stocks were inoculated into 5 mL LB broth and grown overnight at 37°C with shaking at 200 rpm. Culture conditions for RNA-seq samples followed Sanders et al. (7, 16), and Biolog microplate assay conditions are described below.

### Phenotypic profiling using Biolog Gen III microplates

Phenotypic screening was conducted using Biolog Gen III microplates (Biolog Inc., Hayward, CA) to assess metabolic activation across 94 substrates and stress conditions. The assay measures respiratory dye reduction rather than biomass accumulation. Following overnight culture in LB, cells were normalized to an OD_600_ of 0.5 in 12 mL of inoculating fluid A (IF-A), pelleted at 13,200 rpm for 10 min, and resuspended in fresh IF-A. A total of 100 μL of the standardized inoculum was added to each well, and plates were incubated at 37°C with shaking in a Thermo Fisher Varioskan LUX reader. Optical density at 595 nm was recorded hourly for 24 h, and each population was assessed in triplicate. Area under the curve (AUC) values were calculated using empirical trapezoidal numerical integration of measured OD595 time-series data without curve fitting. Error values represent the standard error of the mean across three independent biological replicates. As Biolog Gen III absorbance measurements reflect a composite signal derived from respiratory dye reduction, cellular density, and medium chemistry (including pH-dependent effects), these assays were interpreted as relative indicators of metabolic activation and pathway accessibility rather than direct measurements of biomass accumulation (25). Accordingly, phenotypic comparisons focus on relative differences across strains and conditions rather than absolute growth yields. Statistical differences across populations were assessed using one-way ANOVA with post hoc multiple comparisons of AUC values. AUCs were normalized to WT, and substrates with no statistically significant change were set to 1.0 relative to the WT. This approach preserves relative differences among responsive substrates while preventing non-responsive conditions from artificially inflating phenotypic breadth estimates. These normalized values were then visualized as heatmaps and integrated with previously published genomic and transcriptomic data sets for downstream correlation analyses (7, 16).

### Multivariate analysis of phenotypic data

Principal component analysis (PCA) was performed on normalized AUC values in RStudio (26) using prcomp() with unit variance scaling (scale.=TRUE) on the normalized AUC matrix (strains × conditions). PCA plots were generated using ggplot2, ggrepel, and ggforce, with 95% confidence ellipses to visualize clustering among WT, engineered *cusS* mutants, and SAM populations. Hierarchical clustering was performed using Euclidean distance and Ward’s D2 linkage (27) [hclust(method = “ward.D2”)], and dendrograms were visualized using base plotting functions. Permutational multivariate analysis of variance (PERMANOVA) (27) (adonis2, 999 permutations) was used to test whether R15L-containing strains formed a significantly distinct phenotypic cluster.

### RNA-seq methods

RNA-seq data analyzed in this study were previously published in Sanders et al. (7, 16) and were reprocessed here alongside newly generated phenotypic data. Briefly, total RNA was extracted from mid-log phase cultures using the RNeasy Mini Kit (Qiagen), and libraries were prepared using the NEBNext Ultra II Directional RNA Library Prep Kit. Sequencing was performed on an Illumina NovaSeq 6000 (paired-end 150 bp). Reads were trimmed, aligned to the *E. coli* K-12 MG1655 reference genome (NCBI accession U00096.3), and quantified using HISAT2 (28) and featureCounts (29). Differential expression analysis was carried out in DESeq2 (30) (v1.30.1) with an adjusted *P*-value cutoff of 0.05. Finally, pathway analysis was performed using limma’s (31) “kegg” functionality. The genes that were considered up/down in this analysis were at FDR < 0.05.

### Transcriptomic PCA and multivariate analysis

Raw gene count tables were compiled across all populations and duplicate locus tags were collapsed by summing read counts. Genes with low expression were filtered by retaining those with at least 10 counts in a minimum of two populations to reduce noise from sparsely expressed features. Library size normalization was performed using counts per millions (CPM), followed by log2 transformation.

Principal component analysis (PCA) was conducted on the normalized expression matrix using centered and scaled values to assess global transcriptomic structure among populations. Hierarchical clustering was performed using Euclidean distance and Ward’s minimum variance method on the same normalized data set. Multivariate separation between engineered *cusS* mutants and evolved SAM populations was evaluated using permutational multivariate analysis of variance (PERMANOVA) with 999 permutations.

### Genomic data reuse

Whole-genome sequencing data for SAM populations were previously generated and published in Sanders et al. (16) and were reanalyzed here for consistency with phenotypic and transcriptomic data sets.

### Competitive fitness assays

To determine whether multi-trait phenotypic expansion confers a measurable fitness benefit, competitive fitness assays were performed. Each of the six SAM populations was competed head-to-head against the engineered R15L single mutant in three carbon sources identified as differentially utilized in the Biolog screening: 0.2% sucrose, 0.2% raffinose, and 0.2% sodium butyrate. Overnight cultures of each SAM population and R15L (each with an OD_600_ ~1.7) were co-inoculated at a 1:1 ratio (50 μL of each for a final 1:100 dilution) into Davis Minimal Broth supplemented with the indicated carbon source, then incubated for 24 h at 37°C with shaking. Each competition experiment was completed in triplicate. Following competition, populations were pelleted and genomic DNA was extracted using the Omega Bio-tek E.Z.N.A. Bacterial DNA Kit. Sequencing libraries were prepared using the Illumina DNA Prep kit and sequenced in-house on an Illumina NextSeq 2000. Sequence alignment and variant calling were performed using the breseq 0.39.0 pipeline in polymorphism mode (32) to permit detection of mixed-population allele frequencies, with reads aligned to the *E. coli* K-12 MG1655 reference genome (NC_000913).

Competitor frequency was determined using two independent chromosomal markers. The *cusS* R15L population frequency was tracked using a synonymous mutation in *mdtB* (multidrug efflux pump RND permease subunit MdtB), confirmed by whole-genome sequencing to be fixed in R15L and absent from all six SAM ancestor genomes (17). The *cusS* R15L allele itself was used as a secondary marker where informative: *cusS* is unique to R15L in competitions against SAM1, SAM4, and SAM5 (which do not carry a fixed *cusS* mutation), and its concordance with *mdtB* in these three backgrounds validated *mdtB* as a reliable marker of R15L frequency. However, *cusS* is shared ancestrally between R15L and SAM2, SAM3, and SAM6 and therefore does not discriminate genotype frequency in those three backgrounds. Three biological replicates were performed per strain per environment. Samples were excluded when genome-wide variant counts greatly exceeded the range observed across retained samples (16 to 96 variants in retained samples versus 718 to 2,292 in excluded samples), when sequencing coverage was insufficient for reliable polymorphism calling, or when no sequencing reads were obtained. This criterion was evaluated independently of competitor frequency values. The magnitude of the excess is larger than mixing of a small number of SAM genotypes would produce and is consistent with contamination introduced during sample processing rather than within the competition cultures. Retained replicates per strain and condition are reported in Table S2.

## RESULTS

Silver tolerance of the SAM lineages and single *cusS* mutants was established in prior work (7, 16) and is not re-evaluated here; therefore, the results focus on phenotypic, transcriptomic, and genomic patterns beyond the original selection environment from those initial studies.

### Global phenotypic profiling distinguishes engineered single mutants from evolved lineages

**Fig 1:**
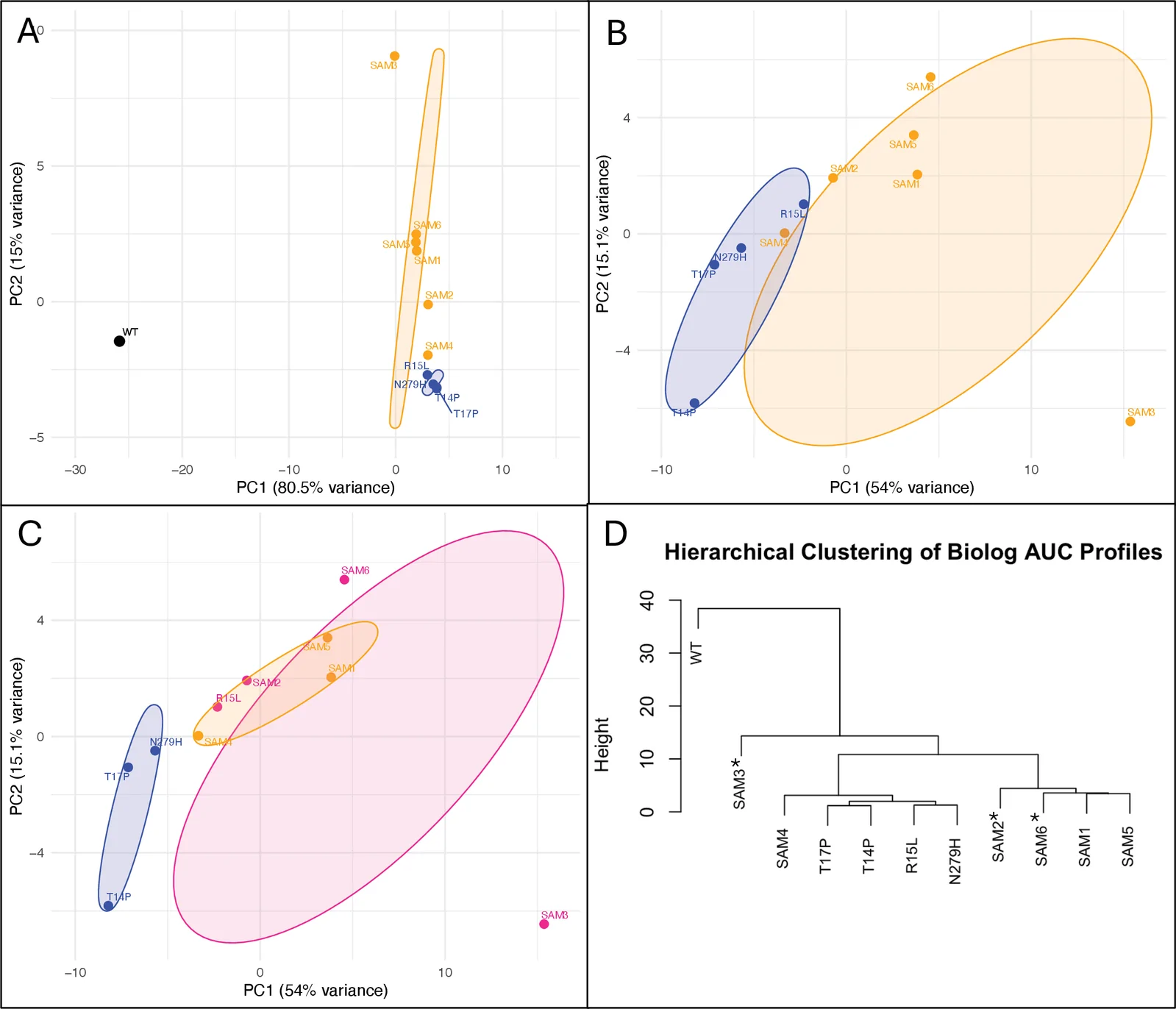
Global divergence in metabolic phenotype space among mutant classes. Principal component analysis (PCA) of normalized Biolog phenotypes illustrating phenotypic separation between engineered *cusS* single mutants and evolved silver-adapted mutant (SAM) populations. (**A**) PCA including WT, engineered mutants, and SAM strains. (**B**) PCA excluding the WT ancestor to visualize separation among mutant classes independent of ancestral baseline effects. (**C**) PCA of R15L-containing strains comparing engineered R15L single mutants and evolved SAM backgrounds, showing separation between engineered and evolved strains carrying the same *cusS* allele. (**D**) Hierarchical clustering based on Euclidean distances illustrating phenotypic similarity relationships among strains. Asterisks (*) indicate SAM strains carrying the *cusS* R15L mutation.

To assess differences in global functional phenotype profiles across strains, principal component analysis (PCA) was performed on normalized Biolog Gen III substrate utilization and stress-response data. PCA revealed clear separation between the ancestral WT strain, engineered *cusS* single mutants, and evolved silver-adapted mutant (SAM) strains (Fig. 1A). When all strains were included, the first two principal components explained 95.5% of total variance (PC1 = 80.5%, PC2 = 15%), reflecting the strong separation of the WT background relative to mutant populations. Engineered mutants clustered tightly in phenotype space, whereas SAM strains exhibited substantially greater dispersion across principal component axes.

When the WT ancestor was excluded from the analysis, separation between engineered *cusS* mutants and SAM strains remained evident (Fig. 1B), with the first two principal components explaining 69.1% of total variance (PC1 = 54.0%, PC2 = 15.1%), indicating that phenotypic divergence reflects differences among mutant classes rather than baseline differences relative to the ancestral background. Using the same ordination shown in Fig. 1B, we restricted the data set to R15L-containing strains, direct comparison of the engineered R15L single mutant with evolved SAM backgrounds carrying the same *cusS* allele (hereafter indicated with an asterisk: SAM2*, SAM3*, and SAM6*) again revealed clear separation between engineered and evolved strains (Fig. 1C), with the first two principal components explaining 69.1% of total variance (PC1 = 54.0%, PC2 = 15.1%). In this subset analysis, strains carrying the R15L mutation did not cluster together, indicating that the *cusS* mutation alone is insufficient to reproduce the phenotypic patterns observed in evolved lineages.

Hierarchical clustering produced a pattern consistent with the PCA results, with engineered *cusS* mutants clustering together and SAM strains forming multiple lineage-specific branches (Fig. 1D). PERMANOVA supported significant separation between engineered *cusS* single mutants and evolved SAM strains (Euclidean distance; F = 8.77, R² =0.523, *P* = 0.013; 999 permutations), indicating that evolved lineages occupy a distinct region of phenotypic space relative to engineered mutants. In contrast, PERMANOVA did not support significant clustering of R15L-containing strains relative to other non-WT strains (Euclidean distance; F = 1.61, R² =0.167, *P* = 0.247; 999 permutations), once again, indicating that the presence of the *cusS* R15L allele alone does not by itself account for observed phenotypic divergence.

### Phenotypic profiles differ between engineered mutants and evolved lineages across multiple trait classes

**Fig 2:**
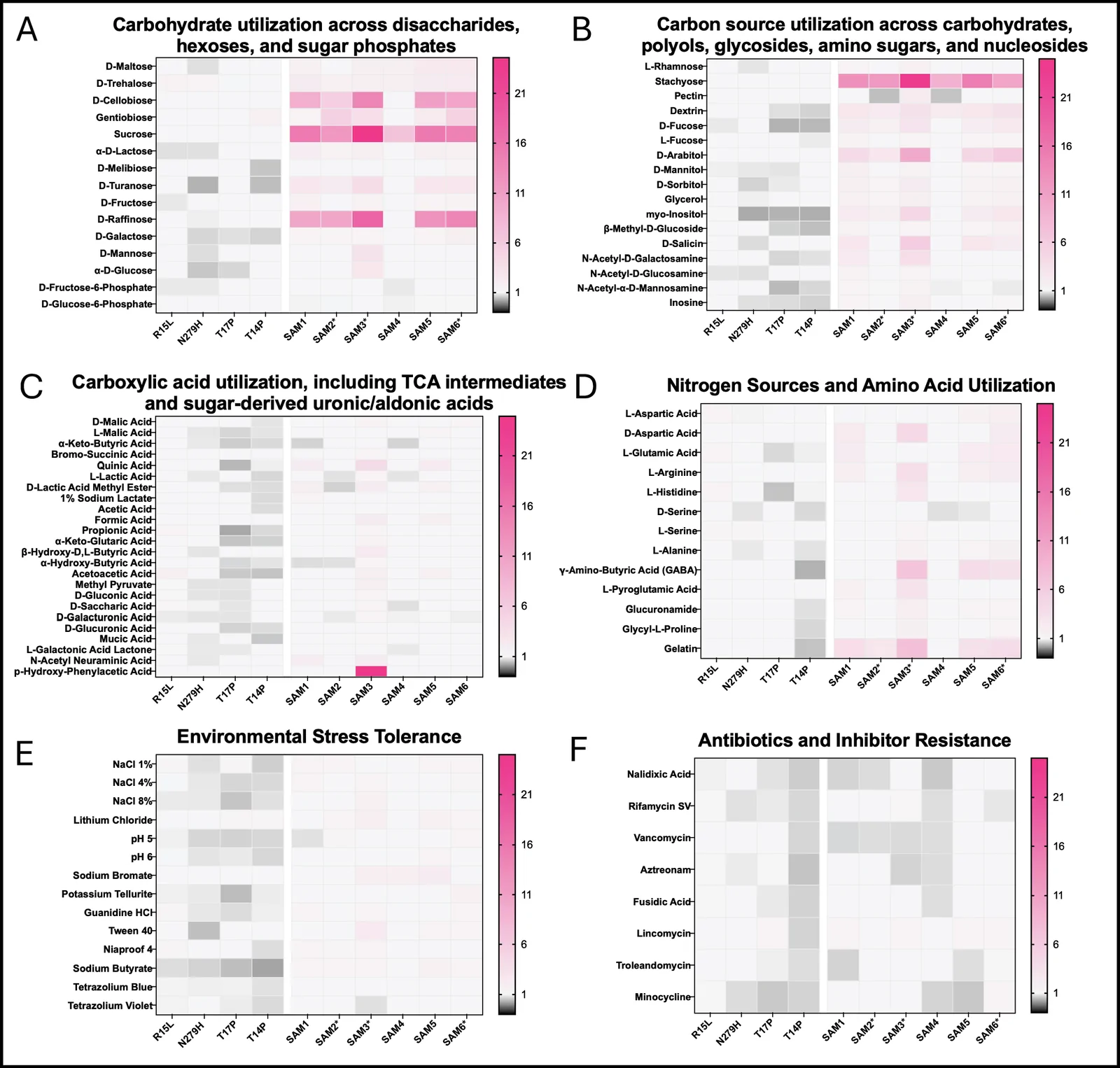
Pleiotropic enhancement across metabolic and stress-response conditions. Heatmaps display normalized area under the curve (AUC) values derived from Biolog respiratory activity assays for engineered *cusS* single mutants and evolved SAM strains. Values are normalized to the WT reference strain (WT = 1.0) and represent relative substrate utilization and stress-response profiles across tested conditions. Panels show responses across (**A**) disaccharides, hexoses, and sugar phosphates; (**B**) additional carbon sources, including polyols and glycosides, (**C**) carboxylic acids, (**D**) nitrogen and amino acid sources, (**E**) environmental stress conditions, and (**F**) antibiotics and inhibitory compounds. Asterisks (*) indicate SAM strains carrying the *cusS* R15L mutation.

To resolve the trait-level patterns underlying the global phenotypic separation observed in Fig. 1, we compared normalized Biolog profiles for engineered *cusS* mutants and evolved SAM strains across multiple metabolic and stress-response categories (Fig. 2). Normalized Biolog profiles revealed consistent differences in substrate utilization and stress tolerance between engineered *cusS* single mutants and evolved SAM strains across multiple condition classes. All phenotypic measurements represent the mean of three independent biological replicates per strain and condition. Representative substrate utilization curves with replicate error bars used for AUC calculations are shown in Fig. S1 to illustrate the temporal response patterns underlying normalized AUC values and to demonstrate the consistency of replicate measurements across substrates exhibiting strong phenotypic divergence, moderate responses, and near-baseline behavior.

Engineered single mutants showed limited changes across most substrates, with generally reduced or near-baseline responses across carbon, nitrogen, stress, and antibiotic conditions (Fig. 2A through F). The R15L single mutant exhibited modest increases on a small subset of nitrogen substrates (Fig. 2D) and under selected stress and inhibitor conditions (Fig. 2E and F) but did not display coordinated multi-category shifts comparable to evolved populations.

In contrast, SAM strains displayed broader variation in phenotype profiles across multiple tested categories. All SAM lineages, except for SAM4, showed increased utilization across multiple carbohydrate substrates spanning disaccharides, hexoses, and sugar phosphates (Fig. 2A), as well as increased utilization of select additional carbon sources including polyols and glycosides (Fig. 2B). Changes in utilization of carboxylic acids, including several TCA-associated and sugar-derived substrates, were not evident except for in SAM3* (Fig. 2C).

Moderate increases were observed for nitrogen and amino acid utilization (Fig. 2D) across all strains except for SAM4. SAM strains also exhibited altered tolerance response profiles under osmotic and chemical stress conditions (Fig. 2E) and both single mutants and SAM populations showed a decreased, compound-specific response across antibiotic and inhibitor conditions (Fig. 2F).

Importantly, phenotypic expansion was not uniform across evolved lineages and across panels, SAM strains exhibited higher normalized AUC values across a greater number of responsive conditions compared with engineered mutants, consistent with increased phenotypic breadth across metabolic and stress-response trait categories. Together, these results indicate that evolved SAM strains display broader multi-category phenotypic expansion than engineered *cusS* single mutants.

### Transcriptomic divergence between engineered and evolved populations

**Fig 3:**
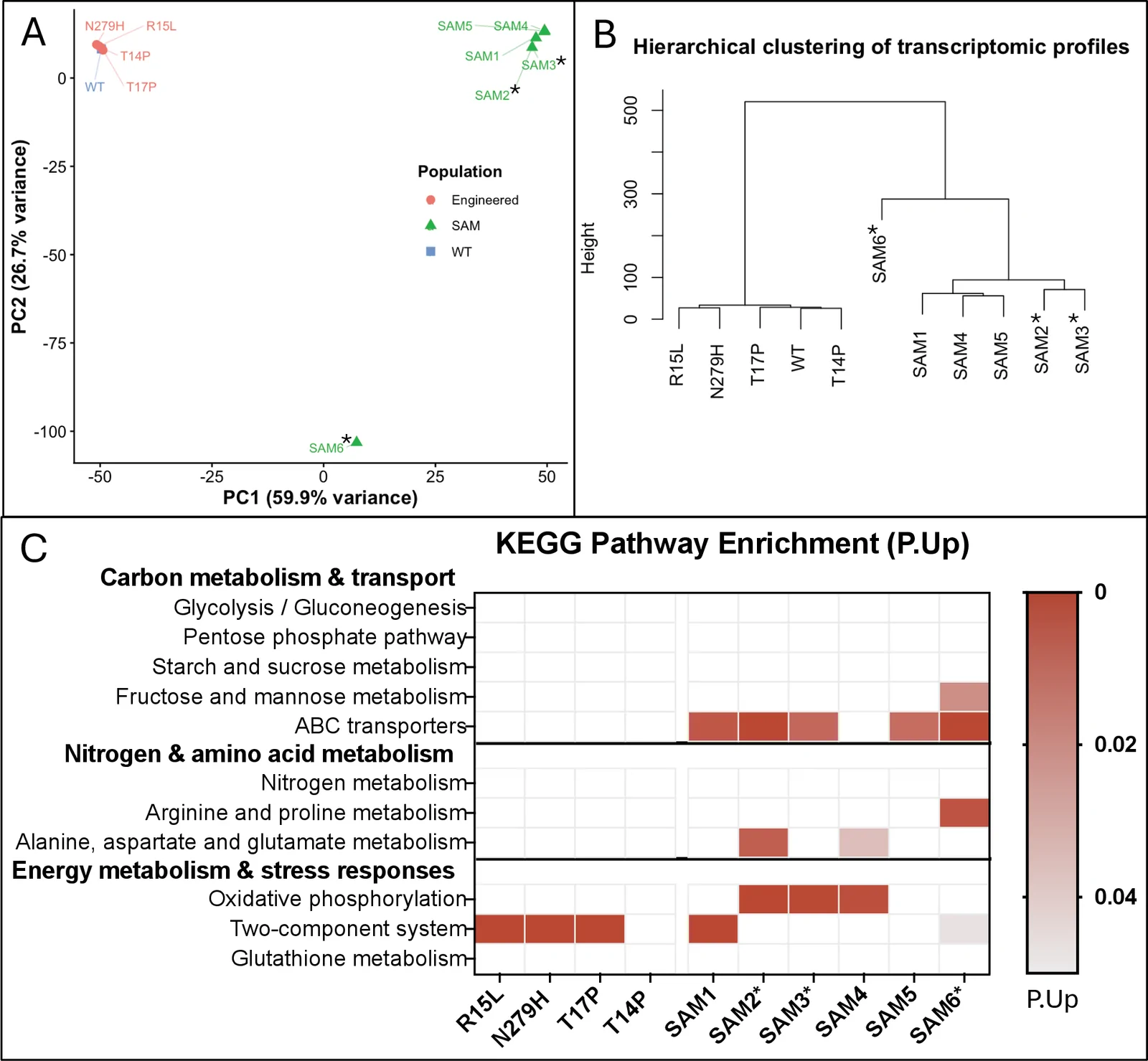
Genome-wide transcriptomic divergence between engineered and evolved strains. (**A**) Principal component analysis (PCA) of CPM-normalized, log₂-transformed RNA-seq expression profiles showing clustering of engineered *cusS* single mutants with the WT strain and separation of evolved SAM populations in expression space. Percent variance explained by each principal component is indicated on the axes. (**B**) Hierarchical clustering of normalized transcriptomic profiles based on Euclidean distance and Ward’s minimum variance method, illustrating grouping relationships among engineered and evolved populations. (**C**) KEGG pathway enrichment heatmap displaying significantly upregulated functional categories (P.Up) across populations. Pathways are grouped by functional category (carbon metabolism and transport, nitrogen and amino acid metabolism, and energy metabolism and stress response). Color intensity reflects enrichment significance, with darker colors indicating stronger enrichment (lower P.Up values). Pathways with enrichment P.Up ≥ 0.05 are shown as background (not significantly enriched). Asterisks (*) indicate SAM strains carrying the *cusS* R15L mutation.

To determine whether the phenotypic differences observed in Fig. 2 were associated with global shifts in gene expression, we performed PCA on normalized genome-wide transcriptomic profiles from engineered *cusS* single mutants, evolved SAM populations, and the ancestral WT strain (Fig. 3A) originally generated in our previous work (7, 16). PCA was conducted using normalized count data to enable direct inclusion of the WT strain in the ordination. PCA revealed clear separation between engineered mutants and evolved SAM populations, with engineered strains clustering tightly with WT, indicating broadly similar global expression profiles. In contrast, SAM populations occupied a distinct region of principal component space, consistent with transcriptomic divergence associated with experimental evolution.

Among evolved lineages, SAM1–SAM5 clustered proximally along PC1, whereas SAM6* exhibited additional separation along PC2 (Fig. 3A), consistent with both the lineage-specific phenotypic heterogeneity observed in Fig. 2 and the distinct positioning of SAM6* relative to other evolved lineages in phenotype space in Fig. 1. The first two principal components explained 86.6% of total variance (PC1 = 59.9%, PC2 = 26.7%), indicating strong global structure in expression profiles across populations.

Hierarchical clustering of normalized expression data produced a pattern consistent with the PCA results, with engineered mutants clustering together and SAM populations forming a distinct branch (Fig. 3B). This class-level separation between engineered and evolved strains is consistent with the phenotypic clustering patterns observed in Fig. 1D. Within the evolved group, SAM6* branched separately from SAM1–SAM5, reflecting greater transcriptomic divergence among evolved lineages. PERMANOVA further supported strong separation between engineered and evolved transcriptomic profiles (Euclidean distance; F = 18.04, R² =0.693, *P* = 0.008; 999 permutations), confirming that evolved populations exhibit a distinct global expression state relative to engineered single mutants.

### Pathway-level transcriptional enrichment distinguishes evolved SAM populations from engineered mutants

To determine whether the global transcriptomic divergence observed in Fig. 3A and B was associated with differences in functional pathway engagement, we performed KEGG pathway enrichment analysis on differentially expressed genes from engineered *cusS* single mutants and evolved SAM populations (Fig. 3C). Engineered single mutants exhibited minimal enrichment relative to evolved SAM populations, across most functional categories, with enrichment largely limited to two-component regulatory system (TCRS) pathways encompassing genes associated with the CusS system (*cusSR* and *cusCFBA*).

In contrast, SAM populations displayed broader enrichment across multiple functional modules. Within carbon metabolism and transport categories, several SAM lineages exhibited significant enrichment of sugar utilization and transport pathways. SAM6* upregulated fructose and mannose metabolism, and all SAM populations except SAM4 upregulated ABC transporter systems, consistent with the expanded substrate utilization capacity observed phenotypically (Fig. 2). Nitrogen and amino acid metabolism pathways were also selectively enriched in subsets of SAM populations (SAM2*, SAM4, and SAM6*), indicating diversification of nutrient-associated functional profiles across evolved lineages. In addition, enrichment of energy metabolism and stress-response pathways, including oxidative phosphorylation (SAM2*, SAM3*, and SAM4) and two-component regulatory systems (SAM1 and SAM6*), was observed, reflecting engagement of both metabolic and regulatory functional categories.

Importantly, as with the Biolog data, enrichment patterns were not uniform across evolved lineages. Individual SAM populations exhibited distinct combinations of enriched pathways, indicating that transcriptional remodeling occurred through multiple lineage-specific configurations rather than a single convergent expression profile. Together, these pathway-level patterns demonstrate that evolved SAM populations exhibit expanded and diversified functional transcriptional states relative to engineered single mutants, providing a transcriptomic framework consistent with the broader and more heterogeneous phenotypic responses observed in Fig. 2.

### Mutational landscape of SAM strains reveals convergent targeting of regulatory and functional pathway nodes

Previously generated whole-genome sequencing (16) data from evolved SAM populations were reanalyzed to examine mutation distributions in relation to the phenotypic and transcriptomic patterns observed in this study (Table 1). Across lineages, mutations were distributed across regulatory genes, global transcriptional control factors, and genes annotated to metabolic and stress-response functional categories, rather than being restricted to a single metabolic pathway or enzyme class. Full variant annotations, including genomic position, nucleotide change, allele frequency, and predicted coding impact, are provided in Table S1.

**TABLE 1:** Distribution of regulatory and functional category-associated mutations across evolved SAM populations^*a*^

Pop.	Freq	Gene	Mutation	Description	Variant type	Category
R15L	1	*cusS <-*	R15L (CGC->CTC)	Sensory histidine kinase in two-component regulatory system with CusR, senses copper ions	SNP	Silver stress sensor/regulatory gateway
SAM2*	1	*cusS <-*	R15L (CGC->CTC)	Sensory histidine kinase in two-component regulatory system with CusR, senses copper ions	SNP	Silver stress sensor/regulatory gateway
SAM3*	1	*cusS <-*	R15L (CGC->CTC)	Sensory histidine kinase in two-component regulatory system with CusR, senses copper ions	SNP	Silver stress sensor/regulatory gateway
SAM4	0.055	*cusS <-*	R15L (CGC->CTC)	Sensory histidine kinase in two-component regulatory system with CusR, senses copper ions	SNP	Silver stress sensor/regulatory gateway
SAM6*	1	*cusS <-*	R15L (CGC->CTC)	Sensory histidine kinase in two-component regulatory system with CusR, senses copper ions	SNP	Silver stress sensor/regulatory gateway
SAM2*	1	*ompR <-*	coding (524/720 nt)	Response regulator in two-component regulatory system with EnvZ	Indel/IS	Two-component/envelope regulation
SAM3*	1	*ompR <-*	coding (524/720 nt)	Response regulator in two-component regulatory system with EnvZ	Indel/IS	Two-component/envelope regulation
SAM6*	1	*ompR <-*	R182C (CGC->TGC)	Response regulator in two-component regulatory system with EnvZ	SNP	Two-component/envelope regulation
SAM1	0.877	*rpoC ->*	T1054N (ACC->AAC)	RNA polymerase, beta prime subunit	SNP	Global regulation/stress
SAM2*	1	*rho ->*	R66C (CGT->TGT)	Transcription termination factor	SNP	Global regulation/stress
SAM3*	1	*rho ->*	R66C (CGT->TGT)	Transcription termination factor	SNP	Global regulation/stress
SAM5	0.164	*rpoB ->*	E142G (GAG->GGG)	RNA polymerase, beta subunit	SNP	Global regulation/stress
SAM6*	1	*rho ->*	G63V (GGT->GTT)	Transcription termination factor	SNP	Global regulation/stress
SAM2*	0.93	*cyaA ->*	S528* (TCG->TAG)	Adenylate cyclase	SNP	Carbon metabolism and transport
SAM2*	0.058	*malF <-*	R112H (CGC->CAC)	Maltose transporter subunit	SNP	Carbon metabolism and transport
SAM3*	0.865	*cyaA ->*	S528* (TCG->TAG)	Adenylate cyclase	SNP	Carbon metabolism and transport
SAM5	0.235	*cyaA ->*	coding (1436-1442/2547 nt)	Adenylate cyclase	SNP	Carbon metabolism and transport
SAM5	0.129	*yhfA <-/- > crp*	intergenic (-148/-150)	OsmC family protein/cAMP-activated global transcription factor, mediator of catabolite repression	Indel/IS	Carbon metabolism and transport
SAM6*	0.141	*cyaA ->*	coding (1436-1442/2547 nt)	Adenylate cyclase	SNP	Carbon metabolism and transport
SAM1	0.178	*gltP ->/<- yjcO*	intergenic (+266/+376)	Glutamate/aspartate:proton symporter/Sel1 family TPR-like repeat protein	SNP	Nitrogen and amino acid metabolism
SAM2*	0.282	*gltP ->/<- yjcO*	intergenic (+266/+376)	Glutamate/aspartate:proton symporter/Sel1 family TPR-like repeat protein	SNP	Nitrogen and amino acid metabolism
SAM2*	0.059	*gltD ->*	R308H (CGC->CAC)	Glutamate synthase, 4Fe-4S protein, small subunit	SNP	Nitrogen and amino acid metabolism
SAM2*	0.052	*gabD ->*	A170A (GCG->GCA)	Succinate-semialdehyde dehydrogenase I, NADP-dependent	SNP	Nitrogen and amino acid metabolism
SAM3*	0.163	*gltD ->*	R308H (CGC->CAC)	Glutamate synthase, 4Fe-4S protein, small subunit	SNP	Nitrogen and amino acid metabolism
SAM3*	0.159	*gltP ->/<- yjcO*	intergenic (+266/+376)	Glutamate/aspartate:proton symporter/Sel1 family TPR-like repeat protein	SNP	Nitrogen and amino acid metabolism
SAM4	0.258	*glnE <-*	coding (1604-1609/2841 nt)	Fused deadenylyltransferase/adenylyltransferase for glutamine synthetase	Indel/IS	Nitrogen and amino acid metabolism
SAM4	0.235	*gltP ->/<- yjcO*	intergenic (+266/+376)	Glutamate/aspartate:proton symporter/Sel1 family TPR-like repeat protein	SNP	Nitrogen and amino acid metabolism
SAM5	0.408	*glnE <-*	E783V (GAA->GTA)	Fused deadenylyltransferase/adenylyltransferase for glutamine synthetase	SNP	Nitrogen and amino acid metabolism
SAM5	0.236	*gltP ->/<- yjcO*	intergenic (+266/+376)	Glutamate/aspartate:proton symporter/Sel1 family TPR-like repeat protein	SNP	Nitrogen and amino acid metabolism
SAM6*	0.318	*lysC <-*	R198L (CGT->CTT)	Lysine-sensitive aspartokinase 3	SNP	Nitrogen and amino acid metabolism
SAM6*	0.226	*gltD ->*	coding (264/1419 nt)	Glutamate synthase, 4Fe-4S protein, small subunit	Indel/IS	Nitrogen and amino acid metabolism
SAM6*	0.193	*gltP ->/<- yjcO*	intergenic (+266/+376)	Glutamate/aspartate:proton symporter/Sel1 family TPR-like repeat protein	SNP	Nitrogen and amino acid metabolism
SAM2*	1	*dnaK ->*	R167G (CGT->GGT)	Chaperone Hsp70, with co-chaperone DnaJ	SNP	Energy metabolism and stress response
SAM3*	1	*dnaK ->*	R167G (CGT->GGT)	Chaperone Hsp70, with co-chaperone DnaJ	SNP	Energy metabolism and stress response
SAM4	0.094	*cyoA <-*	N172S (AAC->AGC)	Cytochrome o ubiquinol oxidase subunit II	SNP	Energy metabolism and stress response
SAM6*	1	*fur <-*	K9N (AAG->AAT)	Ferric iron uptake regulon transcriptional repressor; autorepressor	SNP	Energy metabolism and stress response
SAM1	1	*glnH <-*	A17V (GCG->GTG)	Glutamine transporter subunit	SNP	Other
SAM1	0.849	*yeaH ->*	N10K (AAC->AAA)	UPF0229 family protein	SNP	Other
SAM1	0.807	*pyrE <-/<- rph*	intergenic (−41/+25)	Orotate phosphoribosyltransferase/ribonuclease PH (defective); enzyme; Degradation of RNA; RNase PH	Indel/IS	Other
SAM2*	1	*yggN <-*	I106I (ATC->ATA)	DUF2884 family putative periplasmic protein	SNP	Other
SAM2*	1	*yghS <-*	A166V (GCA->GTA)	Putative ATP-binding protein	SNP	Other
SAM2*	1	*[yicC]*		[yicC]	Indel/IS	Other
SAM2*	1	*yicO <-*	I81R (ATA->AGA)	Putative adenine permease	SNP	Other
SAM3*	1	*yggN <-*	I106I (ATC->ATA)	DUF2884 family putative periplasmic protein	SNP	Other
SAM3*	1	*yghS <-*	A166V (GCA->GTA)	Putative ATP-binding protein	SNP	Other
SAM3*	1	*[yicC]*		[yicC]	Indel/IS	Other
SAM3*	1	*yicO <-*	I81R (ATA->AGA)	Putative adenine permease	SNP	Other
SAM6*	1	*rpoA <-*	V282L (GTA->TTA)	RNA polymerase, alpha subunit	SNP	Other

^*a*^ Asterisks (*) indicate silver adapted strains that carry the R15L mutation.

SAM2*, SAM3*, and SAM6* carried fixed *cusS* R15L mutations, whereas other lineages (SAM1, SAM4, and SAM5) lacked fixed *cusS* alleles or carried only low-frequency variants (Table 1). Of these *cusS*-fixed lineages, SAM3* and SAM6* exhibited the strongest phenotypic and transcriptomic divergence, whereas SAM2* did not display comparable divergence (Fig. 1 and 3). In addition to *cusS*, these *cusS*-fixed SAM populations also accumulated mutations in multiple global and regulatory loci. All three *cusS*-fixed lineages carried mutations affecting the EnvZ/OmpR two-component regulatory system (*ompR*) and transcription termination factor *rho*, while SAM6* additionally carried a mutation in the iron-responsive regulator *fur*. These patterns indicate repeated targeting of regulatory control nodes across independently evolved lineages.

Beyond regulatory loci, SAM populations accumulated mutations in genes annotated to carbon metabolism and transport, nitrogen and amino acid metabolism, and energy metabolism and stress-response categories. Variants in the global carbon signaling regulator *cyaA* were detected across multiple SAM lineages, including SAM2*, SAM3*, SAM5, and SAM6*, while additional mutations affecting carbon transport-associated genes, such as *malF* and *crp*-associated intergenic regions, were observed in select populations. Nitrogen and amino acid metabolism-associated genes, including *gltP*, *gltD*, *glnE*, and *lysC*, were mutated across multiple SAM populations, with individual strains carrying distinct combinations of these variants. Similarly, mutations annotated to energy metabolism and stress-response functions, including *dnaK*, *cyoA*, and *fur*, were distributed across several evolved lineages.

Importantly, mutation patterns were not uniform across SAM populations. Individual lineages accumulated unique combinations of regulatory and functional category-associated variants rather than sharing an identical mutational profile. SAM populations with fewer regulatory mutations, including SAM1 and SAM4, also exhibited fewer variants mapping to transcriptome-enriched functional categories and displayed more limited phenotypic and transcriptomic divergence relative to SAM3* and SAM6*. Together, these results demonstrate that evolved SAM populations exhibit partially overlapping but lineage-specific mutation architectures characterized by repeated targeting of regulatory control nodes and accumulation of variants across transcriptome-aligned functional categories, paralleling the phenotypic and transcriptional divergence patterns observed in this study.

### Competitive fitness against R15L is strain-specific and does not uniformly track with phenotypic breadth

**Fig 4:**
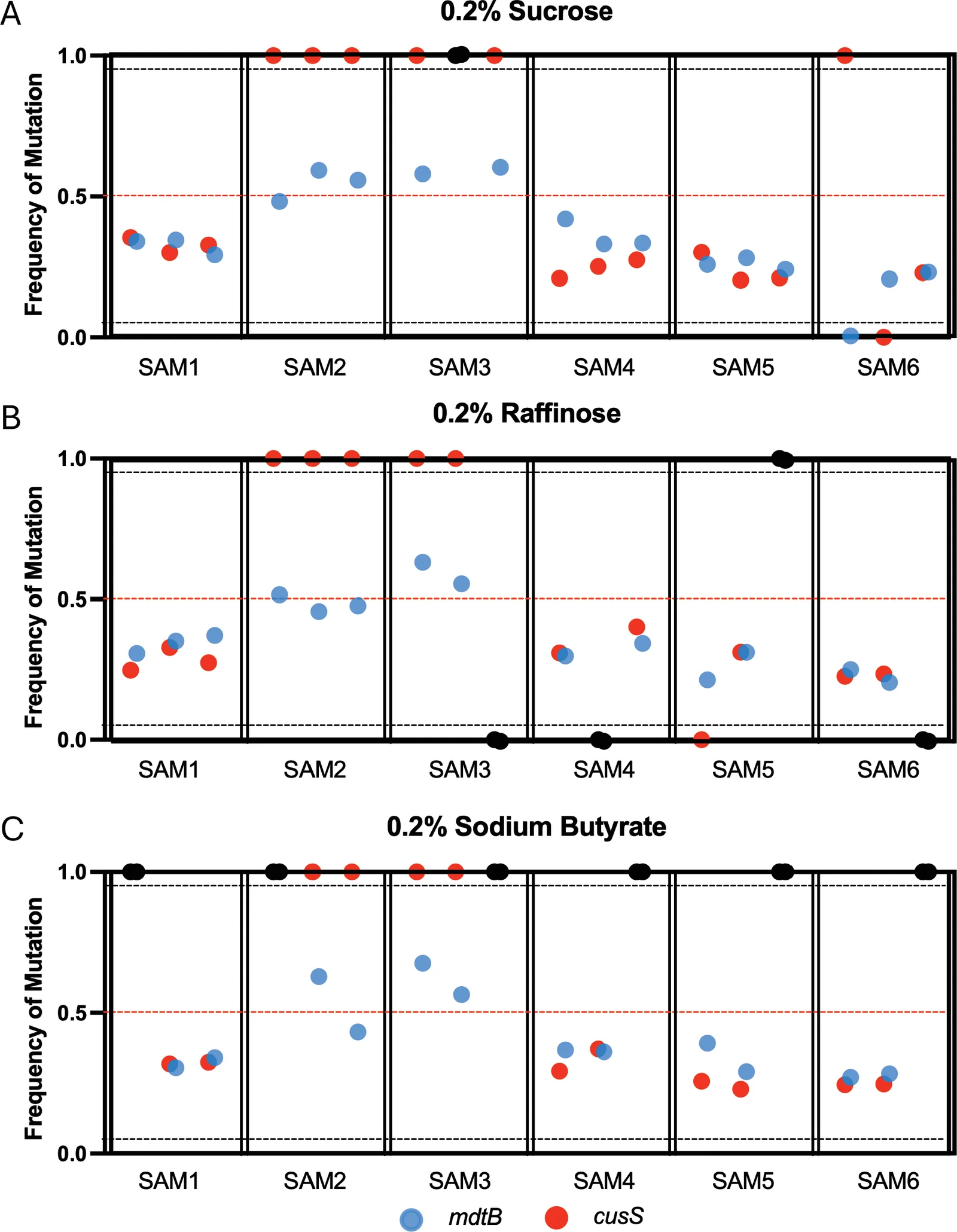
Competitive fitness of silver-adapted mutant (SAM) lineages against the engineered R15L single mutant across three carbon environments. SAM1–SAM6 were each competed head-to-head against R15L in (**A**) 0.2% sucrose, (**B**) 0.2% raffinose, and (**C**) 0.2% sodium butyrate for 24 h, and competitor frequency was determined by whole-genome resequencing. R15L frequency was tracked using *mdtB* (blue), a synonymous mutation confirmed by whole-genome sequencing to be fixed in R15L and absent from all six SAM ancestor genomes. *cusS* (red) is shown as a secondary marker; *cusS* is informative for tracking R15L frequency in competitions against SAM1, SAM4, and SAM5, which do not carry a fixed *cusS* mutation, but is shared ancestrally between R15L and SAM2*, SAM3*, and SAM6* and therefore does not discriminate genotype frequency in those three backgrounds. Black points indicate replicates excluded from analysis owing to elevated genome-wide variant counts or insufficient sequencing coverage. Retained replicates per strain and condition are reported in Table S2 and range from two to three. Black dashed lines indicate the upper and lower limits of reliable variant detection (95% and 5% frequency, respectively); the red dashed line marks equal representation of the two competitors (50:50).

To determine whether the multi-trait phenotypic expansion observed in SAM populations corresponds to a measurable fitness benefit, each SAM lineage was competed head-to-head against the engineered R15L single mutant in three carbon sources identified as differentially utilized in the Biolog screening (0.2% sucrose, 0.2% raffinose, 0.2% sodium butyrate). R15L frequency in all competitions was tracked using *mdtB*, a synonymous mutation confirmed by whole-genome sequencing to be fixed in R15L and absent from every SAM ancestor genome (SAM1 through SAM6), making it a reliable marker of R15L frequency regardless of competitor identity. The *cusS* R15L allele was included as a secondary marker where informative, since it is unique to R15L in competitions against SAM1, SAM4, and SAM5, though shared ancestrally with R15L in SAM2*, SAM3*, and SAM6* (Fig. 4).

Competitive outcome varied by lineage. SAM1, SAM4, and SAM5, the three lineages that did not carry a fixed *cusS* mutation, fell below parity with R15L in every retained competition (SAM1, 0.29 to 0.38, mean 0.33, *n* = 8; SAM4, 0.30 to 0.42, mean 0.35, *n* = 7; SAM5, 0.22 to 0.39, mean 0.28, *n* = 7). SAM6* also fell below parity in every retained competition (0.20 to 0.28 in six replicates, with R15L undetected in the seventh; mean 0.21, *n* = 7). Each lineage was assessed against R15L in all three carbon sources, with two to three replicates per condition (Table S2). These values indicate the direction of competitive outcome rather than the magnitude of the fitness difference.

SAM2* and SAM3* did not fall below parity, though the two differed. SAM2* showed no directional outcome, with R15L frequency ranging from 0.43 to 0.63 across replicates (mean 0.52, *n* = 8). For SAM3*, R15L held a majority in every retained replicate, with frequencies ranging from 0.56 to 0.68 (mean 0.60, *n* = 6).

This result is notable in light of the Biolog data, where SAM3* showed the greatest relative substrate utilization advantage over R15L of any lineage tested in sucrose (Fig. 2A), yet did not translate that advantage into a competitive edge under direct competition in any of the three carbon sources tested.

Whole-genome resequencing of post-competition populations also indicated that *cusS* was not retained uniformly among the three ancestrally *cusS*-fixed lineages. *cusS* remained at its ancestral fixed frequency (*f* = 1.0) in every SAM2* and SAM3* replicate examined. In SAM6*, by contrast, *cusS* frequency declined from its ancestral fixed state to 0.20 to 0.28 in five of the six replicates for which *cusS* frequency could be determined, closely tracking the independent R15L-specific marker *mdtB* in the same samples (mean absolute difference = 0.02), consistent with loss of the *cusS* allele specifically from within the SAM6* lineage.

Together, these results suggest that multi-trait phenotypic expansion is not uniformly associated with a competitive advantage. Four of six evolved lineages fell below parity against R15L and two did not, indicating that phenotypic breadth alone does not reliably predict competitive outcome.

## DISCUSSION

**Fig 5:**
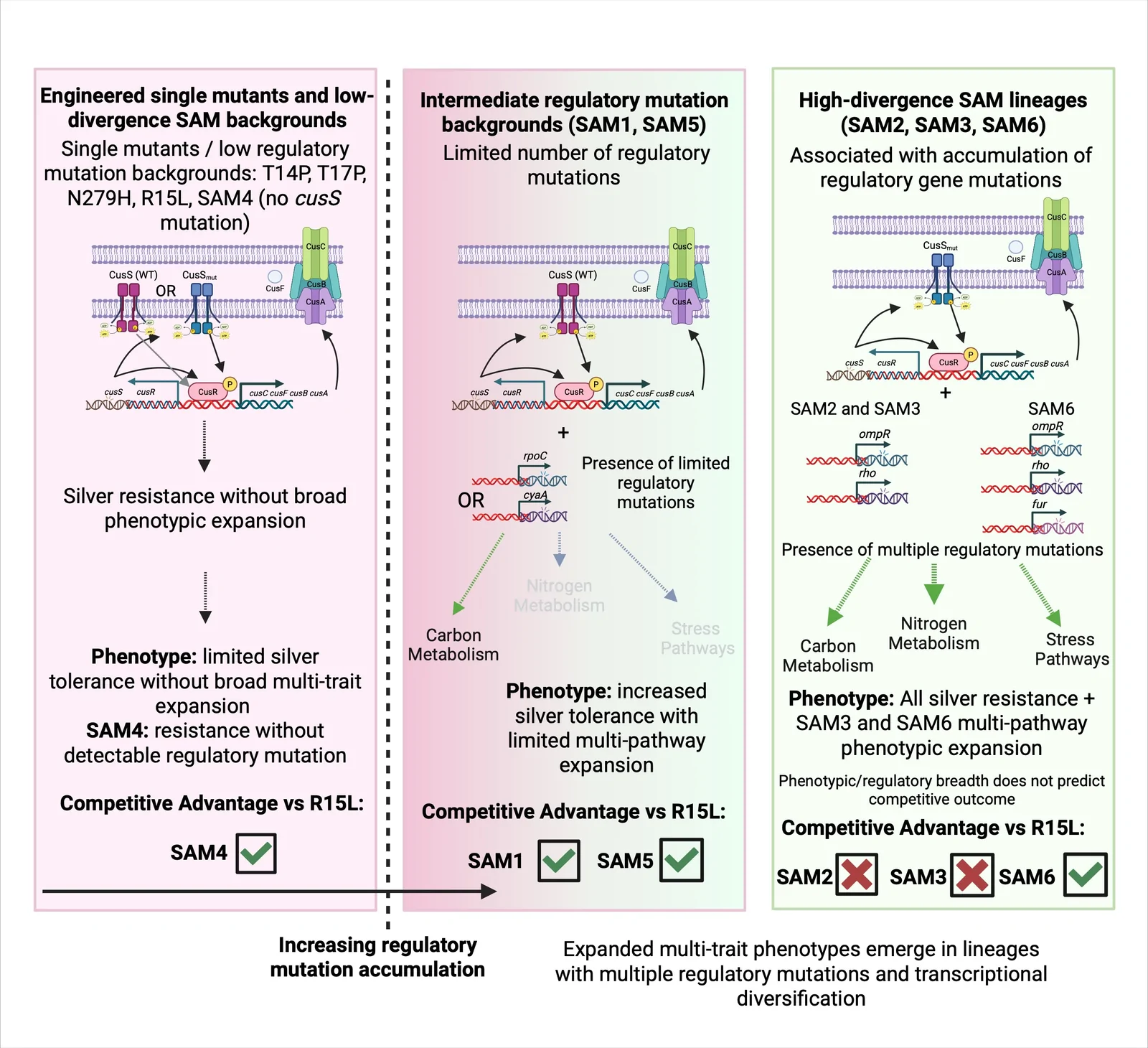
Conceptual summary of regulatory mutation accumulation, multi-trait phenotypic expansion, and competitive fitness outcomes. Schematic illustrating how accumulation of regulatory gene mutations in evolved SAM populations is associated with transcriptional diversification and broader multi-trait phenotypic responses relative to engineered *cusS* single mutants. Competitive advantage against the engineered R15L single mutant (Fig. 4) is indicated for each SAM lineage; phenotypic and regulatory breadth did not reliably predict competitive outcome, as SAM2* and SAM3* failed to outcompete R15L despite carrying regulatory mutations comparable to or exceeding those of lineages that did. This framework summarizes patterns observed across phenotypic, transcriptomic, genomic, and competitive fitness analyses and is intended as a conceptual model rather than a directly tested mechanistic pathway. Created with BioRender.com.

Across phenotypic, transcriptomic, and genomic analyses, evolved SAM lineages consistently differed from engineered single mutants in both trait breadth and greater accumulation of regulatory gene mutations (Fig. 1 to 3; Table 1). As summarized in the conceptual framework presented in Fig. 5, which integrates regulatory mutation accumulation, transcriptomic remodeling, and multi-trait phenotypic expansion, SAM populations exhibiting greater accumulation of regulatory gene mutations, particularly, SAM3* and SAM6*, displayed broader phenotypic expansion across additional trait classes. These patterns indicate that multi-trait phenotypic expansion in SAM populations is associated with regulatory context rather than isolated single adaptive mutations, consistent with broader models of regulatory architecture shaping adaptive potential (15, 33).

This association is supported by replication across independently evolved lineages. Experimental evolution studies have long used replicate populations to infer pleiotropic responses and distinguish them from background-dependent fitness effects by examining the repeatability of trait outcomes across independent evolutionary trajectories (5, 34–37). Classical quantitative genetic theory similarly predicts that correlated responses across multiple traits can arise from pleiotropic genetic covariance rather than independent trait evolution (38). When multiple replicate populations evolving under the same selective environment repeatedly exhibit coordinated changes across multiple traits, the probability that such outcomes arise solely from random genetic background effects becomes increasingly small (5, 39). While direct reconstruction of regulatory mutations in identical genetic backgrounds would provide additional mechanistic resolution, the repeated emergence of similar multi-trait expansion patterns across independent SAM lineages provides inferential support for a network-level interpretation of pleiotropic outcomes rather than lineage-specific idiosyncratic effects (20, 21).

The contrasting behavior of engineered single mutants and evolved SAM lineages further highlights the importance of regulatory context in shaping genotype–phenotype relationships. Engineered mutants carrying isolated *cusS* mutations exhibited limited transcriptional remodeling and constrained phenotypic responses, whereas SAM populations with greater accumulation of regulatory gene mutations displayed broader transcriptional and phenotypic diversification. This pattern is consistent with models in which regulatory interactions introduce strong context dependence, such that the phenotypic consequences of individual mutations depend on network state (23, 24, 40). Under these conditions, identical mutations can yield divergent outcomes depending on regulatory background, contributing to reduced predictability at the single-locus level of genotype–phenotype mapping (39, 41).

This distinction is further supported by phenotypic differences in silver tolerance between engineered mutants and evolved SAM populations observed in our previous work. While SAM populations exhibit robust resistance to inhibitory silver concentrations (7, 19), engineered *cusS* single mutants do not display equivalent resistance relative to the WT background (16, 18). Instead, *cusS* mutants show modest fitness advantages only under sublethal silver exposure. This pattern suggests that *cusS* mutations alone provide partial adaptive benefit but are insufficient to generate full resistance phenotypes, consistent with a multi-step adaptive process in which additional regulatory and network-level modifications contribute to the emergence of high-level silver resistance in evolved populations.

A central question raised by the phenotypic and transcriptomic patterns described above is whether multi-trait expansion in SAM populations reflects a competitive advantage or instead reflects dysregulated network behavior with limited adaptive value outside the original silver selection environment. To address this directly, we compared each SAM lineage against the engineered R15L single mutant in three carbon sources identified as differentially utilized in Biolog profiling. The results did not support a uniform interpretation. SAM1, SAM4, SAM5, and SAM6* each fell below parity with R15L in every retained competition across the three carbon sources tested, indicating that the regulatory mutations carried by these lineages are associated with a competitive advantage under the conditions tested rather than an expanded but non-functional phenotypic repertoire. SAM2* and SAM3*, by contrast, did not show a competitive advantage: SAM2* fluctuated around parity with R15L, and SAM3* showed a modest competitive disadvantage despite exhibiting phenotypic breadth comparable to SAM6*. Taken together, these results suggest that multi-trait phenotypic expansion is not uniformly adaptive: for four of six evolved lineages, expanded phenotypic capacity corresponded to a measurable competitive benefit, while for two, it did not.

The behavior of SAM6* during competition offers a potential mechanistic window into this pattern. Whole-genome resequencing of post-competition SAM6* populations showed that the *cusS* R15L allele, fixed in the ancestral population, declined in frequency in a manner consistent with loss from the SAM6* lineage in most replicates, while SAM6*’s other defining regulatory mutations remained detectable. *cusS* constitutively activates the *cusCFBA* copper/silver efflux system independent of metal exposure (16), a function specific to silver stress. Constitutive expression of bacterial efflux systems is known to carry measurable metabolic costs in the absence of their cognate substrates, although the magnitude of these costs varies by pump identity and environmental context (42). Maintaining the *cusCFBA* system outside the original silver selective environment likely carries an analogous cost with no offsetting benefit, and its apparent loss specifically within the SAM6* lineage during competition, while other regulatory mutations were retained, is consistent with selection against this cost once silver selection is removed. By contrast, *cusS* remained at its ancestral fixed frequency in every SAM2* and SAM3* replicate examined, indicating that this cost, if present, was not resolved in these two lineages within the timeframe of the assay. Competitive outcomes were assessed with two to three replicates per strain and condition, and are interpreted here as indicating the direction of competitive advantage rather than its magnitude.

The contrasting competitive behavior of SAM2* and SAM3*, however, is not fully explained by their mutational profiles. Reanalysis of whole-genome sequencing data (Table 1) (16) showed that SAM2* and SAM3* carry a nearly identical set of fixed mutations, including *cusS, ompR, rho*, and *dnaK*, yet the two lineages diverged in both phenotypic breadth and competitive outcome against R15L. SAM3* additionally showed a significantly reduced growth rate relative to wild type in silver-free medium in prior work (16), a phenotype not observed in SAM2*, which may partly account for SAM3*’s weaker competitive performance independent of cusS status. This apparent disconnect between monoculture growth measures and competitive performance is consistent with prior work showing that growth curve-derived metrics are often poor predictors of relative fitness in direct competition (43), underscoring the value of direct competition assays over solo-culture measures for evaluating adaptive significance. The basis for this divergence between two lineages with essentially identical fixed genomes remains unresolved and may reflect differences in low-frequency segregating variation, regulatory or transcriptional state differences not captured by DNA sequencing, or stochastic effects arising during independent evolutionary trajectories from a shared genetic starting point. This is consistent with prior work showing that near-identical starting genotypes can diverge substantially in adaptive outcome under identical selection, with initial phenotypic superiority not reliably predicting the more successful evolutionary trajectory (44).

Although evolved SAM lineages collectively exhibited expanded phenotypic breadth relative to engineered mutants, individual populations varied substantially in the magnitude and scope of this expansion. In addition, different populations accumulated distinct combinations of regulatory and pathway-associated variants, indicating that multiple genetic routes can produce comparable functional outcomes. This pattern is consistent with theoretical and empirical models of high-dimensional regulatory landscapes in which functional convergence can arise through alternative mutational trajectories (45–47). Such convergence suggests that selection in this system favors regulatory capacity rather than precise genetic solutions, with repeatability emerging at the level of trait architecture rather than nucleotide identity (33).

These findings have important implications for predicting evolutionary outcomes from genomic data. The limited phenotypic divergence observed in engineered mutants illustrates why predictions based solely on individual adaptive mutations often fail (15, 23). In contrast, greater accumulation of regulatory gene mutations in SAM populations was associated with coordinated multi-trait expansion, suggesting that predictive power may improve when evolutionary analyses focus on regulatory context and patterns of regulatory variant accumulation rather than on single-gene effects (33, 48). However, regulatory context alone was not sufficient to predict competitive outcome: SAM2*, SAM3*, and SAM6* shared a nearly identical core regulatory mutation architecture (*cusS, ompR*, and *rho*, with *fur* additionally fixed in SAM6*), yet diverged substantially in both phenotypic breadth and competitive fitness against R15L, indicating that regulatory mutation accumulation alone does not fully account for downstream adaptive outcomes.

Although this study focuses on CusS/CusR-mediated silver resistance, similar regulatory context–dependent dynamics are likely to operate in other signaling systems. TCRSs frequently function as interfaces between environmental sensing and downstream metabolic and stress-response pathways and are known to exhibit strong evolutionary plasticity (6, 9, 12). Mutations affecting these systems can therefore have disproportionate effects on regulatory network behavior across diverse selective environments (7, 49). We therefore expect regulatory mutation accumulation and associated multi-trait expansion to represent a general feature of microbial adaptation under strong environmental selection (2, 3).

Together, these results demonstrate that multi-trait phenotypic expansion in evolved SAM populations is associated with accumulation of regulatory gene mutations and transcriptional diversification rather than with isolated adaptive mutations, consistent with the integrative framework summarized in Fig. 5. By emphasizing regulatory architecture over single-locus effects, this framework provides a stronger foundation for interpreting and predicting complex adaptive responses in microbial populations.

## Data Availability

Whole-genome sequencing data generated for the competitive fitness assays in this study have been deposited in the NCBI BioProject database under accession number PRJNA1508200, with individual data sets accessible via the SRA using accession numbers SAMN62220025–SAMN62220073. All other sequencing data analyzed in this study have been previously published and are publicly available. Whole-genome sequencing data for the engineered cusS single mutants (R15L, T14P, T17P, N279H) generated via pORTMAGE recombineering are available under BioProject PRJNA869586. RNA-seq data for the engineered single mutants can be accessed through the NCBI BioProject database under accession number PRJNA1047097, with individual data sets available via the SRA using accession numbers SRX22705802–SRX22705821. The full genome and transcriptome sequencing data for the silver-adapted mutant (SAM) strains are available under BioProject PRJNA1160277, with individual data sets accessible via the SRA using accession numbers SAMN43760522–SAMN43760542.

## SUPPLEMENTAL MATERIAL

Figure S1 spectrum.00041-26-s0001.pdf Biolog time-course curves.

Table S1 spectrum.00041-26-s0002.pdf SAM mutant full sequencing.

Table S2 spectrum.00041-26-s0003.pdf Sequencing quality control summary for competition assay samples.

ASM does not own the copyrights to Supplemental Material that may be linked to, or accessed through, an article. The authors have granted ASM a non-exclusive, world-wide license to publish the Supplemental Material files. Please contact the corresponding author directly for reuse.
