# Supplementary material for: Regulatory mutation accumulation drives multi-trait phenotypic expansion in silver-adapted Escherichia coli
Source: Microbiol Spectr. 2026 Oct 12;14(11):e00041-26. doi: 10.1128/spectrum.00041-26 (PMC13729116; doi:10.1128/spectrum.00041-26)
Supplement: Supplementary file 1 [file spectrum.00041-26-s0001.pdf]

Substrates exhibiting the largest normalized AUC differences

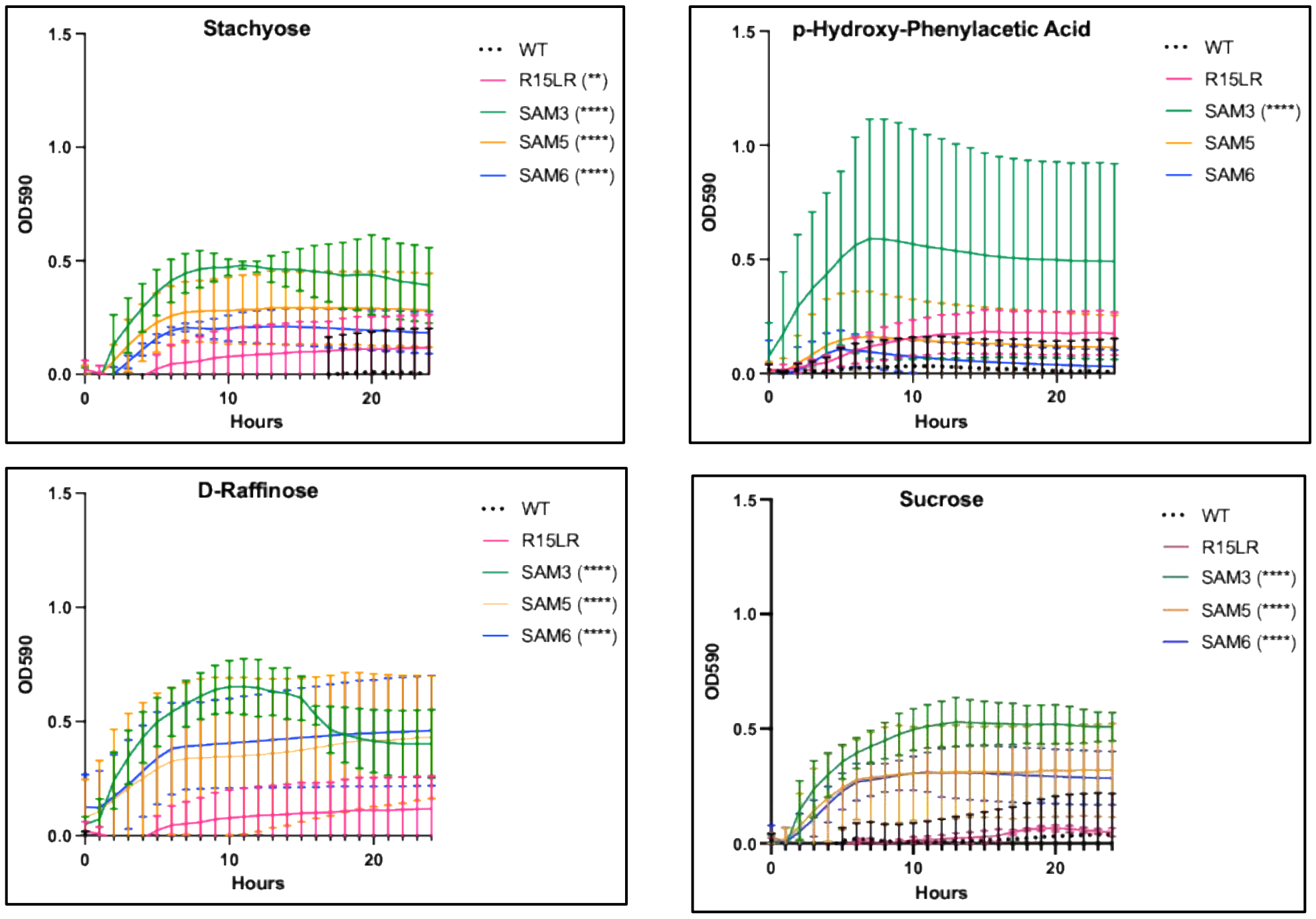

Nitrogen and amino acid utilization substrates

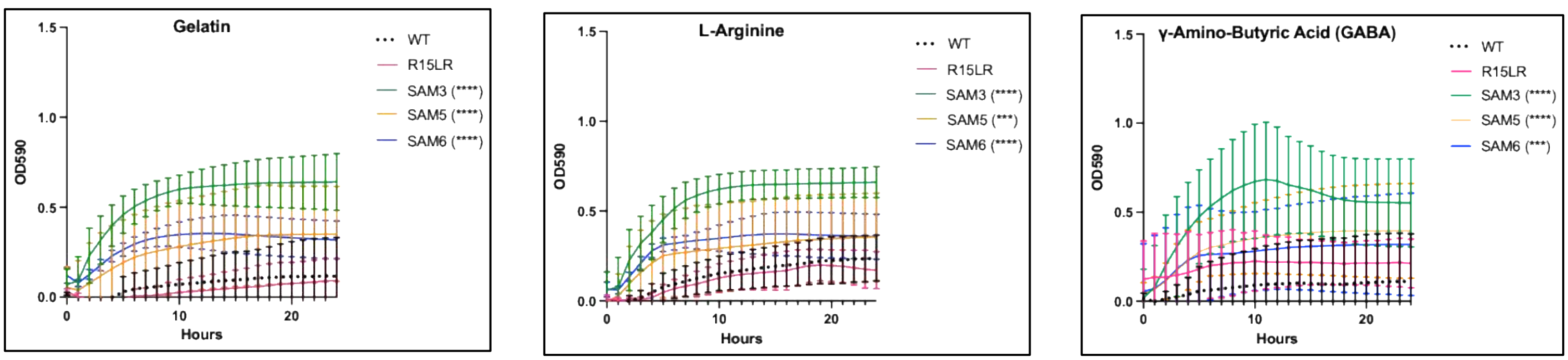

Nitrogen and amino acid utilization substrates

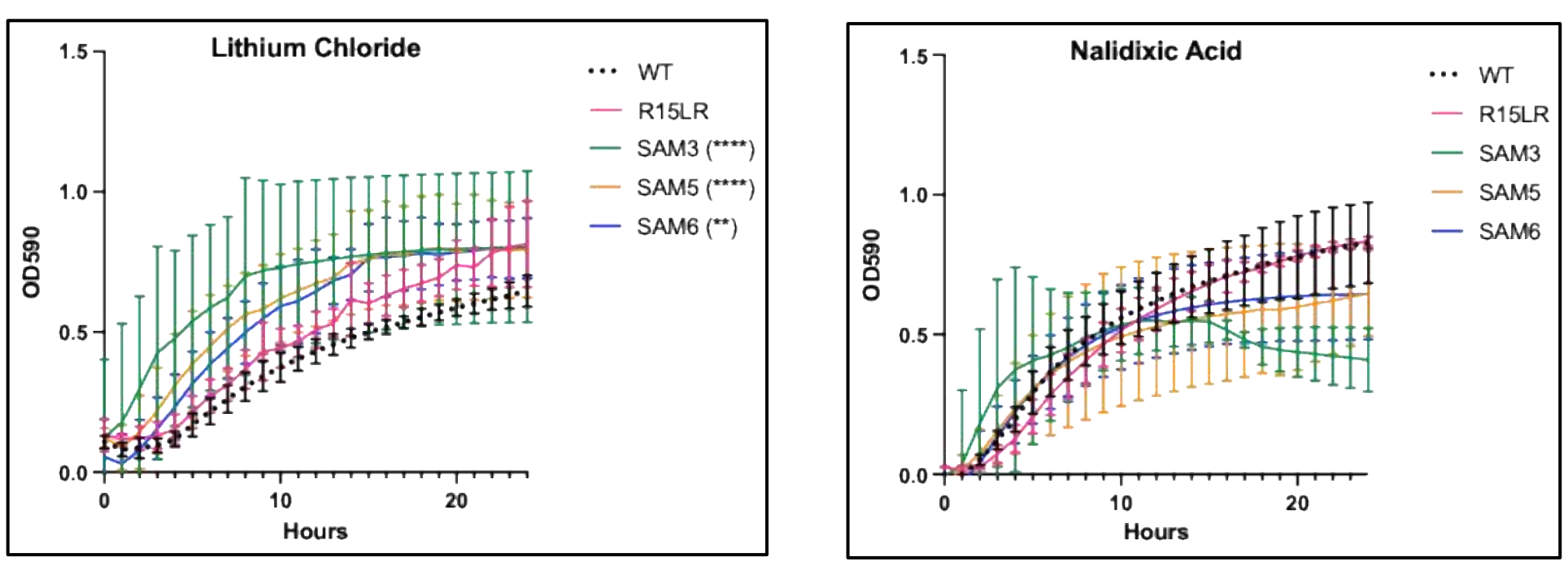

Environmental stress and inhibitor conditions

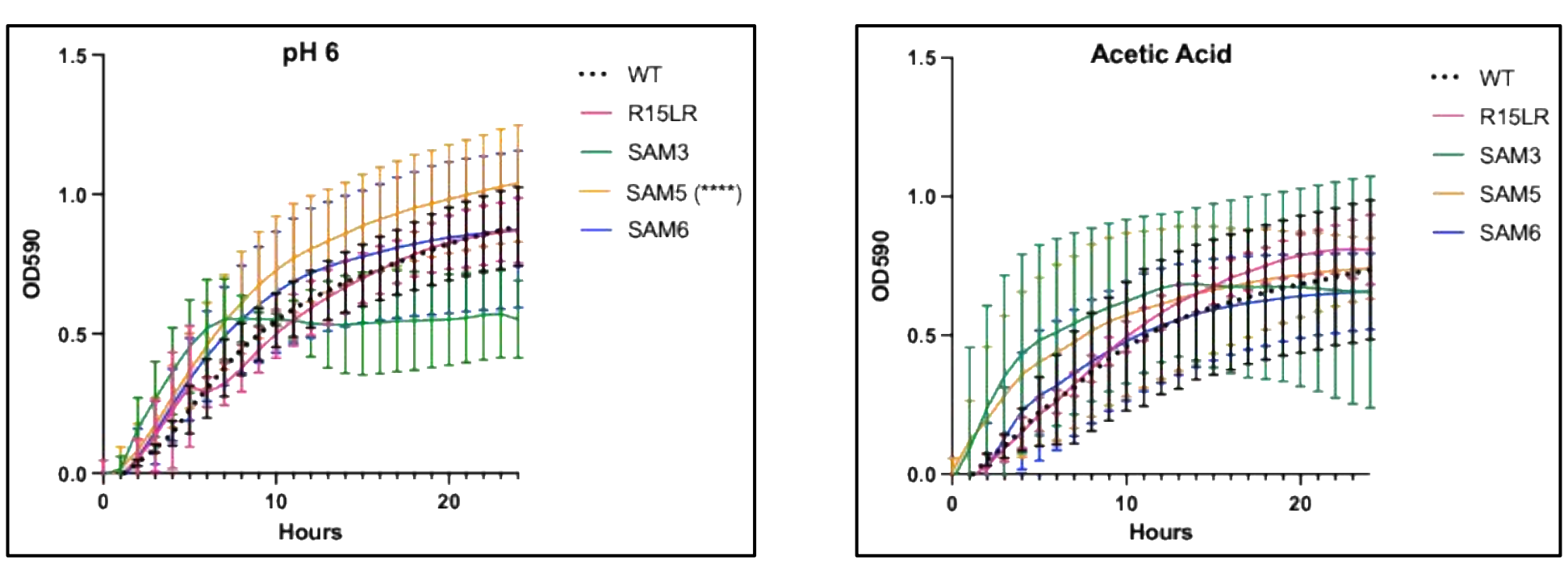

Figure S1. Representative Biolog time-course curves illustrating replicate variability and substrate-specific response patterns

Mean ± SD (n = 3 biological replicates) OD595 time-course curves are shown for representative Biolog Gen III substrates selected to span conditions exhibiting the largest normalized AUC differences, nitrogen and amino acid utilization substrates, environmental stress and inhibitor conditions, and baseline reference conditions with minimal strain-to-strain divergence. These curves correspond to the same measurements used for empirical AUC calculations displayed in Figure 2 and illustrate the consistency of time-course measurements across biological replicates. Together, these panels show that large normalized AUC values reflect sustained separation of respiratory activity trajectories over time rather than isolated measurement outliers.
