# Supplementary material for: Regulatory mutation accumulation drives multi-trait phenotypic expansion in silver-adapted Escherichia coli
Source: Microbiol Spectr. 2026 Oct 12;14(11):e00041-26. doi: 10.1128/spectrum.00041-26 (PMC13729116; doi:10.1128/spectrum.00041-26)
Supplement: Supplementary file 2 [file spectrum.00041-26-s0002.pdf]

Table S1: All mutations present in the silver adapted mutant (SAM) populations

Population	Position	Mutation	Frequency	Gene	Effect	Description	Variant_type	Category
R15L	594727	C->A	1	cusS <-	R15L (CGC->CTC)	sensory histidine kinase in two-component regulatory system with CusR, senses	SNP	CusS/CusR system
SAM1	847955	G->A	1	glnH <-	A17V (GCG->GTG)	glutamine transporter subunit	SNP	Other
SAM1	4188510	C->A	0.877	rpoC ->	T1054N (ACC->AAC)	RNA polymerase, beta prime subunit	SNP	Global regulation / stress
SAM1	1868984	C->A	0.849	yeaH ->	N10K (AAC->AAA)	UPF0229 family protein	SNP	Other
SAM1	3815809	Δ1 bp	0.807	pyrE <- / <- rph	intergenic (-41/+25)	orotate phosphoribosyltransferase/ribonuclease PH (defective);enzyme; Degrad	Indel/IS	Other
SAM1	3831168	C->T	0.732	yicH ->	L238L (CTA->TTA)	putative inner membrane-anchored periplasmic AsmA family protein	SNP	Other
SAM1	4296060	C->T	0.178	gltP -> / <- yjcO	intergenic (+266/+376)	glutamate/aspartate:proton symporter/Sel1 family TPR-like repeat protein	SNP	Nitrogen & amino acid metabolism
SAM1	3047290	A->G	0.153	gcvP <-	R584R (CGT->CGC)	glycine decarboxylase, PLP-dependent, subunit (protein P) of glycine cleavage co	SNP	Other
SAM1	294161	IS2 (+) +5 bp	0.079	yagM <-	coding (635-639/855 nt)	CP4-6 prophage; uncharacterized protein	Indel/IS	Other
SAM1	2067300	C->T	0.075	insH1 <-	G8R (GGA->AGA)	IS5 transposase and trans-activator	SNP	Other
SAM1	1899974	G->A	0.065	yoaD ->	P516P (CCG->CCA)	putative membrane-anchored cyclic-di-GMP phosphodiesterase, regulator of ce	SNP	Other
SAM1	1717681	C->A	0.062	pdxH <-	E109D (GAG->GAT)	pyridoxine 5'-phosphate oxidase	SNP	Other
SAM1	2527378	C->A	0.058	yfeH ->	P145T (CCA->ACA)	putative inorganic ion transporter	SNP	Other
SAM1	375045	G->A	0.057	mhpE -> / -> mhpT	intergenic (+164/-414)	4-hyroxy-2-oxovalerate/4-hydroxy-2-oxopentanoic acid aldolase, class I/3-hyd	SNP	Other
SAM1	1466639	C->T	0.057	ydbA ->	pseudogene (1248/2513 nt)	pseudogene, autotransporter homolog; interrupted by IS2 and IS30	SNP	Other
SAM1	1088656	A->G	0.055	pgaB <-	M401T (ATG->ACG)	poly-beta-1,6-N-acetyl-D-glucosamine (PGA) N-deacetylase outer membrane ex	SNP	Other
SAM1	3264853	C->T	0.051	tdcC <-	M55I (ATG->ATA)	L-threonine/L-serine transporter	SNP	Other
SAM1	2818046	A->G	0.05	argQ <- / <- argZ	intergenic (-186/+13)	tRNA-Arg/tRNA-Arg	SNP	Other
SAM2	12661	C->G	1	dnaK ->	R167G (CGT->GGT)	chaperone Hsp70, with co-chaperone DnaJ	SNP	Energy metabolism & stress response
SAM2	594727	C->A	1	cusS <-	R15L (CGC->CTC)	sensory histidine kinase in two-component regulatory system with CusR, senses	SNP	CusS/CusR system
SAM2	3101306	G->T	1	yggN <-	I106I (ATC->ATA)	DUF2884 family putative periplasmic protein	SNP	Other
SAM2	3133461	G->A	1	yghS <-	A166V (GCA->GTA)	putative ATP-binding protein	SNP	Other
SAM2	3536061	Δ1 bp	1	ompR <-	coding (524/720 nt)	response regulator in two-component regulatory system with EnvZ	Indel/IS	Two-component / envelope regulation
SAM2	3816605	Δ103 bp	1	[yicC]		[yicC]	Indel/IS	Other
SAM2	3843548	A->C	1	yicO <-	I81R (ATA->AGA)	putative adenine permease	SNP	Other
SAM2	3966612	C->T	1	rho ->	R66C (CGT->TGT)	transcription termination factor	SNP	Global regulation / stress
SAM2	3992735	C->A	0.93	cyaA ->	S528* (TCG->TAG)	adenylate cyclase	SNP	Carbon metabolism & transport
SAM2	3177973	IS1 (+) +9 bp	0.552	nudF <- / -> tolC	intergenic (-63/-134)	ADP-ribose pyrophosphatase/transport channel	Indel/IS	Other
SAM2	915226	T->C	0.446	ybjE <- / <- aqpZ	intergenic (-369/+126)	putative transporter/aquaporin Z	SNP	Other
SAM2	2229177	A->G	0.376	yohP -> / <- dusC	intergenic (+112/+261)	uncharacterized protein/tRNA-dihydrouridine synthase C	SNP	Other
SAM2	4296060	C->T	0.282	gltP -> / <- yjcO	intergenic (+266/+376)	glutamate/aspartate:proton symporter/Sel1 family TPR-like repeat protein	SNP	Nitrogen & amino acid metabolism
SAM2	118951	C->T	0.089	ampD ->	S73S (TCC->TCT)	1,6-anhydro-N-acetylmuramyl-L-alanine amidase, Zn-dependent; murein amid	SNP	Other
SAM2	881776	T->C	0.088	dacC ->	V350V (GTT->GTC)	D-alanyl-D-alanine carboxypeptidase (penicillin-binding protein 6a)	SNP	Other
SAM2	1195125	G->A	0.082	icd ->	M1M (ATG->ATA) †	e14 prophage; isocitrate dehydrogenase, specific for NADP+	SNP	Other
SAM2	1441574	C->A	0.077	hslJ <-	G57V (GGT->GTT)	heat-inducible lipoprotein involved in novobiocin resistance	SNP	Other
SAM2	1829888	G->A	0.064	astA <-	A293V (GCC->GTC)	arginine succinyltransferase	SNP	Other
SAM2	2419254	G->A	0.061	yfcE <-	Q179* (CAG->TAG)	phosphodiesterase activity on bis-pNPP	SNP	Other
SAM2	3154991	C->T	0.06	yqhC <-	G76G (GGG->GGA)	transcriptional activator of yqhD	SNP	Other
SAM2	3337061	T->C	0.06	mlaB <-	K66E (AAA->GAA)	ABC transporter maintaining OM lipid asymmetry, cytoplasmic STAS componen	SNP	Other
SAM2	1635859	G->A	0.059	nohQ <-	pseudogene (509/528 nt)	pseudogene, Qin prophage; Phage DNA packaging protein Nu1 family;Phage or P	SNP	Other
SAM2	3360120	G->A	0.059	gltD ->	R308H (CGC->CAC)	glutamate synthase, 4Fe-4S protein, small subunit	SNP	Nitrogen & amino acid metabolism
SAM2	4244741	C->T	0.058	malF <-	R112H (CGC->CAC)	maltose transporter subunit	SNP	Carbon metabolism & transport
SAM2	2493876	G->A	0.056	yfdX <-	A176V (GCT->GTT)	uncharacterized protein	SNP	Other
SAM2	2693690	C->T	0.056	purL <-	T618T (ACG->ACA)	phosphoribosylformyl-glycineamide synthetase	SNP	Other
SAM2	735540	T->C	0.054	rhsO ->	pseudogene (1408/1521 nt)	pseudogene, Rhs family protein	SNP	Other
SAM2	1975163	T->C	0.054	cheA <-	A54A (GCA->GCG)	fused chemotactic sensory histidine kinase in two-component regulatory systen	SNP	Other
SAM2	2201321	C->T	0.054	yehI ->	T348I (ACA->ATA)	DUF4132 domain-containing protein	SNP	Other
SAM2	308177	C->T	0.053	ecpC <-	A386T (GCT->ACT)	ECP production outer membrane protein	SNP	Other
SAM2	1466495	C->T	0.053	ydbA ->	pseudogene (1104/2513 nt)		SNP	Other
SAM2	1466504	A->G	0.053	ydbA ->	pseudogene (1113/2513 nt)	pseudogene, autotransporter homolog; interrupted by IS2 and IS30	SNP	Other
SAM2	1089760	T->C	0.052	pgaB <-	Q33R (CAG->CGG)	poly-beta-1,6-N-acetyl-D-glucosamine (PGA) N-deacetylase outer membrane ex	SNP	Other
SAM2	2110222	A->G	0.052	rfbA <-	V266V (GTT->GTC)	glucose-1-phosphate thymidylyltransferase	SNP	Other
SAM2	2791782	G->A	0.052	gabD ->	A170A (GCG->GCA)	succinate-semialdehyde dehydrogenase I, NADP-dependent	SNP	Nitrogen & amino acid metabolism
SAM2	1003408	T->G	0.051	ycbU ->	Y174D (TAT->GAT)	putative fimbriae protein	SNP	Other
SAM2	2129864	C->T	0.051	wcaD <-	A339T (GCG->ACG)	putative colanic acid polymerase	SNP	Other
SAM2	2967828	G->A	0.051	ptsP <-	L203L (CTG->TTG)	fused PTS enzyme: PEP-protein phosphotransferase (enzyme I)/GAF domain cont	SNP	Other
SAM2	4486493	C->T	0.051	lptF ->	G92G (GGC->GGT)	lipopolysaccharide export ABC permease of the LptBFGC export complex	SNP	Other
SAM2	3571062	A->G	0.05	glgX <-	F86F (TTT->TTC)	glycogen debranching enzyme	SNP	Other
SAM3	12661	C->G	1	dnaK ->	R167G (CGT->GGT)	chaperone Hsp70, with co-chaperone DnaJ	SNP	Energy metabolism & stress response
SAM3	594727	C->A	1	cusS <-	R15L (CGC->CTC)	sensory histidine kinase in two-component regulatory system with CusR, senses	SNP	CusS/CusR system
SAM3	3101306	G->T	1	yggN <-	I106I (ATC->ATA)	DUF2884 family putative periplasmic protein	SNP	Other
SAM3	3133461	G->A	1	yghS <-	A166V (GCA->GTA)	putative ATP-binding protein	SNP	Other
SAM3	3536061	Δ1 bp	1	ompR <-	coding (524/720 nt)	response regulator in two-component regulatory system with EnvZ	Indel/IS	Two-component / envelope regulation
SAM3	3816605	Δ103 bp	1	[yicC]		[yicC]	Indel/IS	Other
SAM3	3843548	A->C	1	yicO <-	I81R (ATA->AGA)	putative adenine permease	SNP	Other
SAM3	3966612	C->T	1	rho ->	R66C (CGT->TGT)	transcription termination factor	SNP	Global regulation / stress
SAM3	3992735	C->A	0.865	cyaA ->	S528* (TCG->TAG)	adenylate cyclase	SNP	Carbon metabolism & transport
SAM3	3177973	IS1 (+) +9 bp	0.648	nudF <- / -> tolC	intergenic (-63/-134)	ADP-ribose pyrophosphatase/transport channel	Indel/IS	Other
SAM3	3360120	G->A	0.163	gltD ->	R308H (CGC->CAC)	glutamate synthase, 4Fe-4S protein, small subunit	SNP	Nitrogen & amino acid metabolism
SAM3	4296060	C->T	0.159	gltP -> / <- yjcO	intergenic (+266/+376)	glutamate/aspartate:proton symporter/Sel1 family TPR-like repeat protein	SNP	Nitrogen & amino acid metabolism
SAM3	2725169	Δ1 bp	0.147	kgtP <-	coding (578/1299 nt)	alpha-ketoglutarate transporter	Indel/IS	Other
SAM3	2229205	G->C	0.116	yohP -> / <- dusC	intergenic (+140/+233)	uncharacterized protein/tRNA-dihydrouridine synthase C	SNP	Other
SAM3	2771529	IS3 (+) +3 bp	0.079	yfjT ->	coding (382-384/468 nt)	CP4-57 prophage; putative periplasmic protein	Indel/IS	Other
SAM3	1667662	A->G	0.07	mlc <-	C301C (TGT->TGC)		SNP	Other
SAM3	2039690	C->T	0.069	yedY ->	A71A (GCC->GCT)	membrane-anchored, periplasmic TMAO, DMSO reductase	SNP	Other
SAM3	2888245	T->C	0.066	cysH <-	E23G (GAA->GGA)	phosphoadenosine phosphosulfate reductase; PAPS reductase, thioredoxin dep	SNP	Other
SAM3	529421	T->C	0.059	ybbD -> / -> ylbI	intergenic (+291/-79)	pseudogene/pseudogene, Rhs family protein	SNP	Other
SAM3	2328469	G->A	0.058	yfaQ <-	S443S (AGC->AGT)	tandem DUF2300 domain protein, putative host defense protein	SNP	Other
SAM3	585179	G->A	0.056	ompT <-	A152V (GCC->GTC)	DLP12 prophage; outer membrane protease VII (outer membrane protein 3b)	SNP	Other
SAM3	4325888	G->T	0.055	yjdN <- / <- yjdM	intergenic (-147/+511)	metalloprotein superfamily protein/zinc-ribbon family protein	SNP	Other
SAM3	2335324	A->G	0.052	yfaA <-	S441P (TCG->CCG)	DUF2138 family protein, putative host defense protein	SNP	Other
SAM3	4092315	C->T	0.052	frvB <-	A18T (GCT->ACT)	putative PTS enzyme, IIB component/IIC component	SNP	Other
SAM3	4261089	G->A	0.051	yjbM ->	G164E (GGG->GAG)	uncharacterized protein	SNP	Other
SAM4	3815859	Δ82 bp	0.533	[rph]–[rph]		[rph], [rph]	Indel/IS	Other
SAM4	3815824	G->T	0.277	pyrE <- / <- rph	intergenic (-56/+10)	orotate phosphoribosyltransferase/ribonuclease PH (defective);enzyme; Degrad	SNP	Other
SAM4	3198033	IS186 (+) +6 bp :: Δ2 bp	0.258	glnE <-	coding (1604-1609/2841 nt)	fused deadenylyltransferase/adenylyltransferase for glutamine synthetase	Indel/IS	Nitrogen & amino acid metabolism
SAM4	4296060	C->T	0.235	gltP -> / <- yjcO	intergenic (+266/+376)	glutamate/aspartate:proton symporter/Sel1 family TPR-like repeat protein	SNP	Nitrogen & amino acid metabolism
SAM4	3815809	Δ1 bp	0.138	pyrE <- / <- rph	intergenic (-41/+25)	orotate phosphoribosyltransferase/ribonuclease PH (defective);enzyme; Degrad	Indel/IS	Other
SAM4	915226	T->C	0.112	ybjE <- / <- aqpZ	intergenic (-369/+126)	putative transporter/aquaporin Z	SNP	Other
SAM4	451096	T->C	0.094	cyoA <-	N172S (AAC->AGC)	cytochrome o ubiquinol oxidase subunit II	SNP	Energy metabolism & stress response
SAM4	1708718	G->A	0.071	rsxC ->	A600A (GCG->GCA)	SoxR iron-sulfur cluster reduction factor component; putative membrane-asso	SNP	Other
SAM4	1869932	IS2 (+) +5 bp	0.064	yeaH ->	coding (978-982/1284 nt)	UPF0229 family protein	Indel/IS	Other
SAM4	3328463	IS4 (–) +11 bp	0.061	greA <-	coding (243-253/477 nt)	transcript cleavage factor	Indel/IS	Other
SAM4	594727	C->A	0.055	cusS <-	R15L (CGC->CTC)	sensory histidine kinase in two-component regulatory system with CusR, senses	SNP	CusS/CusR system
SAM4	1821249	T->C	0.055	chbC <- / <- chbB	intergenic (-35/+50)	N,N'-diacetylchitobiose-specific enzyme IIC component of PTS/N,N'-diacetylchi	SNP	Other
SAM4	2288354	G->A	0.052	yejO <-	pseudogene (561/2525 nt)	pseudogene, autotransporter outer membrane homology;putative transport; N	SNP	Other
SAM5	3197294	T->A	0.408	glnE <-	E783V (GAA->GTA)	fused deadenylyltransferase/adenylyltransferase for glutamine synthetase	SNP	Nitrogen & amino acid metabolism
SAM5	3815809	Δ1 bp	0.373	pyrE <- / <- rph	intergenic (-41/+25)	orotate phosphoribosyltransferase/ribonuclease PH (defective);enzyme; Degrad	Indel/IS	Other
SAM5	3815859	Δ82 bp	0.367	[rph]–[rph]		[rph], [rph]	Indel/IS	Other
SAM5	4296060	C->T	0.236	gltP -> / <- yjcO	intergenic (+266/+376)	glutamate/aspartate:proton symporter/Sel1 family TPR-like repeat protein	SNP	Nitrogen & amino acid metabolism
SAM5	3992588	(ATCAGCC)2->1	0.235	cyaA ->	coding (1436-1442/2547 nt)	adenylate cyclase	SNP	Carbon metabolism & transport
SAM5	1907503	IS3 (–) +5 bp :: +T	0.178	yobF <-	coding (85-89/144 nt)	DUF2527 family heat-induced protein	Indel/IS	Other
SAM5	4181669	A->G	0.164	rpoB ->	E142G (GAG->GGG)	RNA polymerase, beta subunit	SNP	Global regulation / stress
SAM5	3485966	IS2 (+) +5 bp	0.129	yhfA <- / -> crp	intergenic (-148/-150)	OsmC family protein/cAMP-activated global transcription factor, mediator of ca	Indel/IS	Carbon metabolism & transport
SAM5	3897059	C->A	0.129	yieH ->	L96I (CTC->ATC)	phosphoenolpyruvate and 6-phosphogluconate phosphatase	SNP	Other
SAM5	224808	T->C	0.089	rrsH ->	noncoding (1038/1542 nt)	16S ribosomal RNA of rrnH operon	SNP	Other
SAM5	900123	G->A	0.08	artJ <-	V151V (GTC->GTT)	arginine binding protein, periplasmic	SNP	Other
SAM5	293326	A->G	0.071	yagL <-	I198T (ATC->ACC)	CP4-6 prophage; DNA-binding protein	SNP	Other
SAM5	1466210	G->A	0.068	ydbA ->	pseudogene (819/2513 nt)	pseudogene, autotransporter homolog; interrupted by IS2 and IS30	SNP	Other
SAM5	1287037	C->T	0.061	narI -> / <- rttR	intergenic (+511/+29)	nitrate reductase 1, gamma (cytochrome b(NR)) subunit/rtT sRNA, processed fro	SNP	Other
SAM5	1269450	C->T	0.058	rdlA -> / <- ldrB	intergenic (+61/+253)	sRNA antisense regulator affects LdrA translation; proposed addiction module in	SNP	Other
SAM5	1269451	G->T	0.058	rdlA -> / <- ldrB	intergenic (+62/+252)	sRNA antisense regulator affects LdrA translation; proposed addiction module in	SNP	Other
SAM5	1496764	C->T	0.054	ydcJ -> / -> opgD	intergenic (+133/-92)	putative metalloenzyme/osmoregulated periplasmic glucan (OPG) biosynthesis	SNP	Other
SAM5	1466201	T->C	0.051	ydbA ->	pseudogene (810/2513 nt)	pseudogene, autotransporter homolog; interrupted by IS2 and IS30	SNP	Other
SAM6	594727	C->A	1	cusS <-	R15L (CGC->CTC)	sensory histidine kinase in two-component regulatory system with CusR, senses	SNP	CusS/CusR system
SAM6	710620	C->A	1	fur <-	K9N (AAG->AAT)	ferric iron uptake regulon transcriptional repressor; autorepressor	SNP	Energy metabolism & stress response
SAM6	3440186	C->A	1	rpoA <-	V282L (GTA->TTA)	RNA polymerase, alpha subunit	SNP	Other
SAM6	3536041	G->A	1	ompR <-	R182C (CGC->TGC)	response regulator in two-component regulatory system with EnvZ	SNP	Two-component / envelope regulation
SAM6	3966604	G->T	1	rho ->	G63V (GGT->GTT)	transcription termination factor	SNP	Global regulation / stress
SAM6	4232641	C->A	0.318	lysC <-	R198L (CGT->CTT)	lysine-sensitive aspartokinase 3	SNP	Nitrogen & amino acid metabolism
SAM6	1213820	G->C	0.279	bluR <-	D80E (GAC->GAG)	repressor of blue light-responsive genes	SNP	Other
SAM6	3359461	Δ1 bp	0.226	gltD ->	coding (264/1419 nt)	glutamate synthase, 4Fe-4S protein, small subunit	Indel/IS	Nitrogen & amino acid metabolism
SAM6	3815809	Δ1 bp	0.196	pyrE <- / <- rph	intergenic (-41/+25)	orotate phosphoribosyltransferase/ribonuclease PH (defective);enzyme; Degrad	Indel/IS	Other
SAM6	4296060	C->T	0.193	gltP -> / <- yjcO	intergenic (+266/+376)	glutamate/aspartate:proton symporter/Sel1 family TPR-like repeat protein	SNP	Nitrogen & amino acid metabolism
SAM6	1153243	G->T	0.154	fabF -> / -> pabC	intergenic (+63/-57)	3-oxoacyl-[acyl-carrier-protein] synthase II/4-amino-4-deoxychorismate lyase	SNP	Other
SAM6	3992588	(ATCAGCC)2->1	0.141	cyaA ->	coding (1436-1442/2547 nt)	adenylate cyclase	SNP	Carbon metabolism & transport

SAM6	1734295	G->A	0.087	grxD <- / -> mepH	intergenic (-194/-140)	glutaredoxin-4/murein DD-endopeptidase, space-maker hydrolase	SNP	Other
SAM6	1783972	A->G	0.08	fadK ->	T314T (ACA->ACG)	short chain acyl-CoA synthetase, anaerobic	SNP	Other
SAM6	3638173	C->T	0.077	pitA ->	R178C (CGT->TGT)	phosphate transporter, low-affinity; tellurite importer	SNP	Other
SAM6	1529496	T->C	0.074	rhsE ->	pseudogene (1595/2037 nt)	pseudogene, Rhs family protein	SNP	Other
SAM6	1417872	C->T	0.071	ydaE <-	C46Y (TGT->TAT)	Rac prophage; conserved protein	SNP	Other
SAM6	1118073	G->C	0.069	yceI <-	T135R (ACG->AGG)	secreted protein	SNP	Other
SAM6	1406828	C->T	0.066	ydaM <-	R323H (CGC->CAC)	diguanylate cyclase, csgD regulator	SNP	Other
SAM6	1415602	T->A	0.061	recE <-	I595I (ATA->ATT)	Rac prophage; exonuclease VIII, 5' -> 3' specific dsDNA exonuclease	SNP	Other
SAM6	4214313	G->T	0.061	metA ->	V12F (GTC->TTC)	homoserine O-transsuccinylase	SNP	Other
SAM6	913671	C->T	0.06	hcp <-	W48* (TGG->TGA)	hybrid-cluster [4Fe-2S-2O] protein in anaerobic terminal reductases	SNP	Other
SAM6	2480006	G->A	0.06	dsdA ->	D269N (GAT->AAT)	D-serine dehydratase	SNP	Other
SAM6	1900663	C->A	0.059	yoaE <-	G308V (GGT->GTT)	putative membrane protein/conserved protein	SNP	Other
SAM6	777885	C->A	0.058	tolB ->	A49E (GCG->GAG)	periplasmic protein	SNP	Other
SAM6	1136015	C->T	0.058	flgH ->	T151M (ACG->ATG)	flagellar protein of basal-body outer-membrane L ring	SNP	Other
SAM6	1495623	G->A	0.058	ydcJ ->	R112R (CGG->CGA)	putative metalloenzyme	SNP	Other
SAM6	2545513	G->A	0.058	murR <-	Q33* (CAA->TAA)	repressor for murPQ, MurNAc 6-P inducible	SNP	Other
SAM6	2560599	T->C	0.058	yffL ->	L115L (TTG->CTG)	CPZ-55 prophage; uncharacterized protein	SNP	Other
SAM6	2278939	G->T	0.057	bcr <-	R274R (CGC->CGA)	bicyclomycin/cysteine/sulfonamide efflux transporter	SNP	Other
SAM6	3614258	G->A	0.057	nikA ->	D198N (GAT->AAT)	nickel-binding, heme-binding periplasmic protein	SNP	Other
SAM6	372696	C->T	0.056	mhpD ->	S194S (AGC->AGT)	2-keto-4-pentenoate hydratase	SNP	Other
SAM6	1244206	A->G	0.056	ycgR <-	L108L (TTA->CTA)	flagellar velocity braking protein, c-di-GMP-regulated	SNP	Other
SAM6	3915514	C->T	0.056	glmU <- / <- atpC	intergenic (-314/+39)	fused N-acetyl glucosamine-1-phosphate uridyltransferase/glucosamine-1-pho:	SNP	Other
SAM6	4522674	T->C	0.056	yjhG <-	K474R (AAG->AGG)	putative dehydratase	SNP	Other
SAM6	2179710	A->G	0.055	yegT ->	Y297C (TAT->TGT)	nucleoside transporter, low affinity	SNP	Other
