# Supplementary material for: Regulatory mutation accumulation drives multi-trait phenotypic expansion in silver-adapted Escherichia coli
Source: Microbiol Spectr. 2026 Oct 12;14(11):e00041-26. doi: 10.1128/spectrum.00041-26 (PMC13729116; doi:10.1128/spectrum.00041-26)
Supplement: Supplementary file 3 [file spectrum.00041-26-s0003.pdf]

Supplemental Table S2. Sequencing quality control summary for competition assay samples.

Samples were excluded from competitive fitness analysis when genome-wide variant counts greatly exceeded the range observed across retained samples, when sequencing coverage was insufficient for reliable polymorphism calling, or when no sequencing reads were obtained. Retained samples carried 16 to 96 variants, whereas excluded samples carried 718 to 2292, so no sample required a borderline judgment. This criterion was evaluated from alignment and variant-calling output independently of competitor frequency values, and exclusion was therefore not conditioned on the competitive outcome of a replicate.

The magnitude of the excess in flagged samples is larger than mixing of a small number of SAM genotypes would produce, each of which carries on the order of ten mutations (Table 1), and is consistent with contamination introduced during sample processing rather than within the competition cultures. Retained replicates per strain and condition range from two to three.

Environment	Strain	Rep	Sample	Tube #	Variant count	Mean coverage (x)	% reads mapped	Included	Reason for exclusion
0.2% Sucrose	SAM1	1	Sucrose_SAM1_1	1	36	165.9	100	Yes	
0.2% Sucrose	SAM1	2	Sucrose_SAM1_2	7	68	162.2	100	Yes	
0.2% Sucrose	SAM1	3	Sucrose_SAM1_3	13	53	157.5	100	Yes	
0.2% Sucrose	SAM2*	1	Sucrose_SAM2_1	2	25	178.9	100	Yes	
0.2% Sucrose	SAM2*	2	Sucrose_SAM2_2	8	66	115.1	100	Yes	
0.2% Sucrose	SAM2*	3	Sucrose_SAM2_3	14	70	127.7	100	Yes	
0.2% Sucrose	SAM3*	1	Sucrose_SAM3_1	3	57	146.2	100	Yes	
0.2% Sucrose	SAM3*	2	Sucrose_SAM3_2	9	1518	213.5	100	No	Elevated variant count
0.2% Sucrose	SAM3*	3	Sucrose_SAM3_3	15	92	145.8	100	Yes	
0.2% Sucrose	SAM4	1	Sucrose_SAM4_1	4	29	176.3	100	Yes	
0.2% Sucrose	SAM4	2	Sucrose_SAM4_2	10	74	174.7	100	Yes	
0.2% Sucrose	SAM4	3	Sucrose_SAM4_3	16	70	347.9	100	Yes	
0.2% Sucrose	SAM5	1	Sucrose_SAM5_1	5	16	171.9	100	Yes	
0.2% Sucrose	SAM5	2	Sucrose_SAM5_2	11	85	134.3	100	Yes	
0.2% Sucrose	SAM5	3	Sucrose_SAM5_3	17	77	192.1	100	Yes	
0.2% Sucrose	SAM6*	1	Sucrose_SAM6_1	6	29	149.2	100	Yes	
0.2% Sucrose	SAM6*	2	Sucrose_SAM6_2	12	84	194.8	100	Yes	
0.2% Sucrose	SAM6*	3	Sucrose_SAM6_3	18	68	248	100	Yes	
0.2% Raffinose	SAM1	1	Raffinose_SAM1_1	25	59	114.7	100	Yes	
0.2% Raffinose	SAM1	2	Raffinose_SAM1_2	31	75	140.8	100	Yes	
0.2% Raffinose	SAM1	3	Raffinose_SAM1_3	37	54	111	100	Yes	
0.2% Raffinose	SAM2*	1	Raffinose_SAM2_1	26	58	129.7	100	Yes	
0.2% Raffinose	SAM2*	2	Raffinose_SAM2_2	32	56	132.1	100	Yes	
0.2% Raffinose	SAM2*	3	Raffinose_SAM2_3	38	79	167.2	100	Yes	
0.2% Raffinose	SAM3*	1	Raffinose_SAM3_1	27	73	77.7	100	Yes	

Environment	Strain	Rep	Sample	Tube #	Variant count	Mean coverage (x)	% reads mapped	Included	Reason for exclusion
0.2% Raffinose	SAM3*	2	Raffinose_SAM3_2	33	96	110.5	100	Yes	
0.2% Raffinose	SAM3*	3	Raffinose_SAM3_3	39	75	82.6	100	No	Low coverage
0.2% Raffinose	SAM4	1	Raffinose_SAM4_1	28	60	186.1	100	Yes	
0.2% Raffinose	SAM4	2	Raffinose_SAM4_2	34	n.d.	n.d.	n.d.	No	No sequencing reads obtained
0.2% Raffinose	SAM4	3	Raffinose_SAM4_3	40	44	95.2	100	Yes	
0.2% Raffinose	SAM5	1	Raffinose_SAM5_1	29	58	166.8	100	Yes	
0.2% Raffinose	SAM5	2	Raffinose_SAM5_2	35	74	171.8	100	Yes	
0.2% Raffinose	SAM5	3	Raffinose_SAM5_3	41	1130	179	100	No	Elevated variant count
0.2% Raffinose	SAM6*	1	Raffinose_SAM6_1	30	73	135	100	Yes	
0.2% Raffinose	SAM6*	2	Raffinose_SAM6_2	36	84	99.7	100	Yes	
0.2% Raffinose	SAM6*	3	Raffinose_SAM6_3	42	718	137.7	100	No	Elevated variant count
0.2% Sodium butyrate	SAM1	1	SodiumButyrate_SAM1_1	43	1432	234.4	100	No	Elevated variant count
0.2% Sodium butyrate	SAM1	2	SodiumButyrate_SAM1_2	49	65	107.7	100	Yes	
0.2% Sodium butyrate	SAM1	3	SodiumButyrate_SAM1_3	55	75	141.5	100	Yes	
0.2% Sodium butyrate	SAM2*	1	SodiumButyrate_SAM2_1	44	1978	285.9	100	No	Elevated variant count
0.2% Sodium butyrate	SAM2*	2	SodiumButyrate_SAM2_2	50	69	171.1	100	Yes	
0.2% Sodium butyrate	SAM2*	3	SodiumButyrate_SAM2_3	56	68	179.2	100	Yes	
0.2% Sodium butyrate	SAM3*	1	SodiumButyrate_SAM3_1	45	84	154.8	100	Yes	
0.2% Sodium butyrate	SAM3*	2	SodiumButyrate_SAM3_2	51	72	187.2	100	Yes	
0.2% Sodium butyrate	SAM3*	3	SodiumButyrate_SAM3_3	57	1448	224.5	100	No	Elevated variant count
0.2% Sodium butyrate	SAM4	1	SodiumButyrate_SAM4_1	46	65	161.6	100	Yes	
0.2% Sodium butyrate	SAM4	2	SodiumButyrate_SAM4_2	52	64	130.7	100	Yes	
0.2% Sodium butyrate	SAM4	3	SodiumButyrate_SAM4_3	58	1083	160.2	100	No	Elevated variant count

Environment	Strain	Rep	Sample	Tube #	Variant count	Mean coverage (x)	% reads mapped	Included	Reason for exclusion
0.2% Sodium butyrate	SAM5	1	SodiumButyrate_SAM5_1	47	80	160.9	100	Yes	
0.2% Sodium butyrate	SAM5	2	SodiumButyrate_SAM5_2	53	75	163.9	100	Yes	
0.2% Sodium butyrate	SAM5	3	SodiumButyrate_SAM5_3	59	2292	323.4	100	No	Elevated variant count
0.2% Sodium butyrate	SAM6*	1	SodiumButyrate_SAM6_1	48	86	149.3	100	Yes	
0.2% Sodium butyrate	SAM6*	2	SodiumButyrate_SAM6_2	54	77	176.4	100	Yes	
0.2% Sodium butyrate	SAM6*	3	SodiumButyrate_SAM6_3	60	2268	293.7	100	No	Elevated variant count
